# Friction Mediates Scission of Tubular Membranes Scaffolded by BAR Proteins

**DOI:** 10.1016/j.cell.2017.05.047

**Published:** 2017-06-29

**Authors:** Mijo Simunovic, Jean-Baptiste Manneville, Henri-François Renard, Emma Evergren, Krishnan Raghunathan, Dhiraj Bhatia, Anne K. Kenworthy, Gregory A. Voth, Jacques Prost, Harvey T. McMahon, Ludger Johannes, Patricia Bassereau, Andrew Callan-Jones

**Affiliations:** 1 Laboratoire Physico Chimie Curie, Institut Curie, PSL Research University, CNRS UMR168, 75005 Paris, France; 2 Sorbonne Universités, UPMC University Paris 06, 75005 Paris, France; 3 Department of Chemistry, Institute for Biophysical Dynamics, James Franck Institute, The University of Chicago, 5735 S. Ellis Avenue, Chicago, IL 60637, USA; 4 Subcellular Structure and Cellular Dynamics Unit, Institut Curie, PSL Research University, CNRS UMR144, 75005 Paris, France; 5 Chemical Biology of Membranes and Therapeutic Delivery Unit, Institut Curie, PSL Research University, CNRS UMR3666, INSERM U1143, 75005 Paris, France; 6 Medical Research Council Laboratory of Molecular Biology, Francis Crick Avenue, Cambridge CB2 0QH, UK; 7 Centre for Cancer Research and Cell Biology, Queen’s University Belfast, 97 Lisburn Road, Belfast BT9 7BL, UK; 8 Department of Molecular Physiology and Biophysics, Vanderbilt School of Medicine, 718 Light Hall, Nashville, TN 37232, USA; 9 Mechanobiology Institute, National University of Singapore, Singapore 119077, Singapore; 10 Laboratoire Matière et Systèmes Complexes, CNRS UMR7057, 75205 Paris, France

**Keywords:** endocytosis, membrane scission, membrane tube, BAR domain, endophilin, scaffold, friction-driven scission, diffusion barrier, in vitro reconstitution, molecular motors

## Abstract

Membrane scission is essential for intracellular trafficking. While BAR domain proteins such as endophilin have been reported in dynamin-independent scission of tubular membrane necks, the cutting mechanism has yet to be deciphered. Here, we combine a theoretical model, in vitro, and in vivo experiments revealing how protein scaffolds may cut tubular membranes. We demonstrate that the protein scaffold bound to the underlying tube creates a frictional barrier for lipid diffusion; tube elongation thus builds local membrane tension until the membrane undergoes scission through lysis. We call this mechanism friction-driven scission (FDS). In cells, motors pull tubes, particularly during endocytosis. Through reconstitution, we show that motors not only can pull out and extend protein-scaffolded tubes but also can cut them by FDS. FDS is generic, operating even in the absence of amphipathic helices in the BAR domain, and could in principle apply to any high-friction protein and membrane assembly.

## Highlights

• BAR protein scaffolds form a lipid diffusion barrier on membrane nanotubes
• Elongation force on tubes reveals scaffold-membrane friction
• Local tension rises due to friction, leading to pore nucleation and tube scission
• Microtubule-associated molecular motors pull and cut scaffolded tubes

## Graphical Abstract

**Figure:**
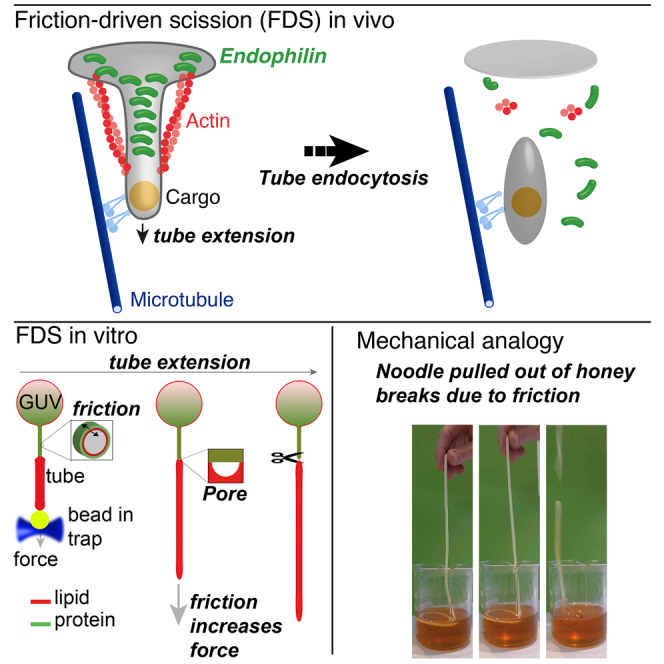

## Teaser

Proteins create points of friction at sites of membrane tubulation leading to scission and vesicle budding.

## Introduction

Endocytosis allows cells to internalize nutrients and proteins and is used by pathogens in the course of infection (McMahon and Boucrot, 2011). While clathrin-mediated endocytosis (CME) has been investigated for many years (Kirchhausen et al., 2014, Merrifield and Kaksonen, 2014, Schmid et al., 2014), clathrin-independent endocytoses (CIEs) have begun to be revealed only recently (Johannes et al., 2015, Soykan et al., 2016). Scission, the process of detachment of the endocytic bud from the plasma membrane, differs between CME and CIE. In CME in mammalian cells, scission requires the assembly of dynamin at the neck of the vesicle, and it can be assisted by actin polymerization (Boulant et al., 2011, Ferguson et al., 2009) and Bin/Amphiphysin/Rvs (BAR) proteins (Meinecke et al., 2013, Neumann and Schmid, 2013, Sundborger et al., 2011, Yoshida et al., 2004). In CIE, which typically involves tubular membrane structures, scission appears to require a more equal division of labor among these three proteins (Renard et al., 2015).

In both CME and CIE, several scission modules may coexist in a single endocytic pathway, rendering the process more robust yet obscuring the underlying mechanisms. In the most prominent scission mechanism, dynamin polymerizes at the neck of the clathrin vesicle or on a tubular tether and then tightens it upon guanosine triphosphate (GTP) hydrolysis until it breaks (Morlot et al., 2012, Roux et al., 2006, Shnyrova et al., 2013). However, scission may take place in the absence of nucleotide hydrolysis. Line tension at the edge of lipid domains can generate enough constriction to drive spontaneous vesiculation or scission of tubes (Allain et al., 2004, Römer et al., 2010). In yeast, it may be assisted by forces exerted by actin polymerization (Liu et al., 2006). Finally, shallow insertion of amphipathic helices (AHs) into the bilayer may lead to scission of small vesicles, as observed in the case of epsin and N-BAR proteins (Boucrot et al., 2012, Simunovic et al., 2013).

Direct in vitro evidence of scission of preformed membrane tubes by N-BAR proteins has not been observed under static conditions, indicating a possible difference between scission mechanisms of spherical and those of tubular membranes. Our in vitro pilot study showed that endophilin A2 (endoA2)-scaffolded tubes undergo scission when extended by an external force in CIE (Renard et al., 2015). Although dynein molecular motors walking on microtubules have been shown to be crucial for tube elongation during CIE in vivo (Day et al., 2015), the scission mechanism and the role of molecular motors remain unknown.

Here, we combine a minimal experimental system and devise a theoretical model to describe this unexplored scission mechanism. We demonstrate that friction between a BAR protein scaffold and an elongated membrane tube increases membrane tension up to tube rupture upon elongation. Our experiments allow us to discriminate our proposed scission model from existing ones, namely, pinching by hemifission or constriction by line tension, thus identifying the minimal components needed to cut membrane tubes stabilized by BAR protein scaffolds. We term the mechanism friction-driven scission (FDS) and demonstrate that motor proteins provide the necessary elongation force in the cell.

## Results

### External Elongation Force Induces Scission of Endophilin-Scaffolded Tubes

**Figure S1:**
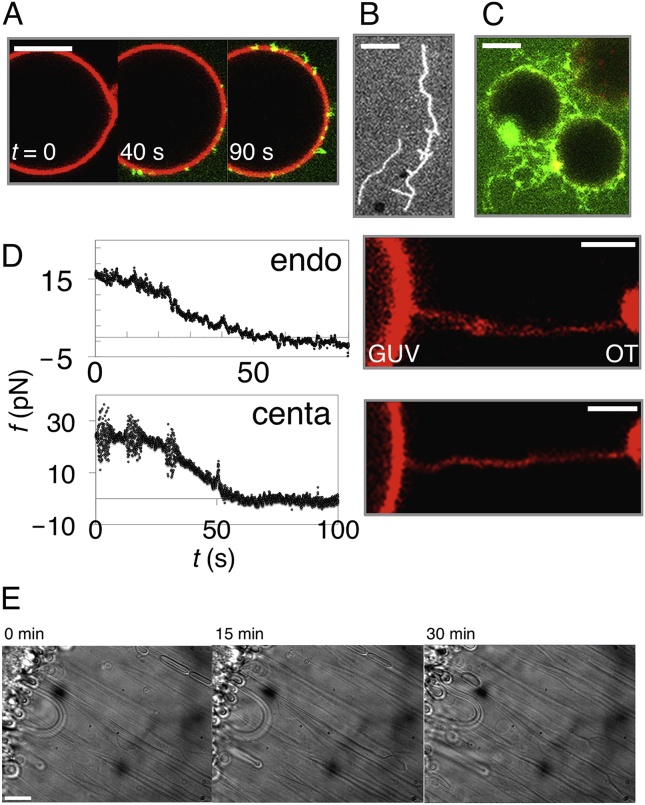
Membrane-Curving Proteins Stabilize Tensed and Tensionless Membrane Tubes and Do Not Cut Them in the Absence of External Force, Related to Figure 1 (A) Spontaneous tubulation of an aspired GUV injected with 1 μM endophilin A2 (monomer concentration in the pipette). Green, endophilin; red, lipids. (B) Spontaneous tubulation of a supported lipid bilayer by 1 μM endophilin A2 (monomer concentration in bulk). Fluorescence, lipids. (C) Spontaneous tubulation of GUVs by 1 μM β2 centaurin (monomer concentration in bulk). Green, centaurin; red, lipids. Scale bar (A–C), 5 μm. (D) Force, *f*, exerted by a membrane tube on a bead held in the optical trap (OT) at time *t* after the injection of 0.5–2.5 μM protein (left) and final confocal microscopy snapshots (right). Tested proteins: endophilin A2 (endo), β2 centaurin (centa). Fluorescence, lipids. Scale bar, 3 μm. (E) Differential interference contrast microscopy time-lapse images of multilamellar bilayer sheets incubated with 5 μM full-length endophilin A2 (monomeric concentration). No change in tubule length or their amount observed in 30 min of imaging time. Membrane composition in (A), (C), (D), and (E): total brain extract + 5% PI(4,5)P_2_ (mol/mol), composition in (B): 30% DOPS, 70% DOPC. Scale bar, 5 μm.

We first studied whether BAR domain proteins may induce scission of flat or tubular membranes. We considered two BAR proteins: endoA2, containing four AHs per dimer, and β2 centaurin (centaurin), containing no AHs. As models of the cell membrane, we created (1) giant unilamellar vesicles (GUVs), (2) tensed supported bilayers, and (3) tensionless membrane sheets. GUVs were composed of the total brain lipid extract supplemented with 5% phosphatidylinositol 4,5-bisphosphate (PI(4,5)P_2_, mol/mol). Consistent with previous findings (Peter et al., 2004, Sorre et al., 2012, Takei et al., 1999), both proteins with 1–5 μM concentrations induced spontaneous tubulation of all three membrane systems, with no evidence of scission (Figure S1; Movie S1). To see whether BAR proteins cut cylindrical membranes, mimicking tubular membrane transport intermediates, we pulled membrane nanotubes from GUVs using optical tweezers. The tube-pulling force *f* can be measured from the movement of the bead in the optical trap, while the tube radius *r* and vesicle tension $\sigma_{\text{v}}$ can be controlled by micropipette aspiration (see STAR Methods). Under a range of $\sigma_{\text{v}}$ (0.001–0.4 mN.m^−1^) and *r* (10–120 nm), we did not observe tube scission in most cases (Table S1). Instead, these proteins stabilized membrane tubes by forming a scaffold, as previously described (Simunovic et al., 2016) and as evidenced by the reduction in tube force (Figure S1).

**Figure 1:**
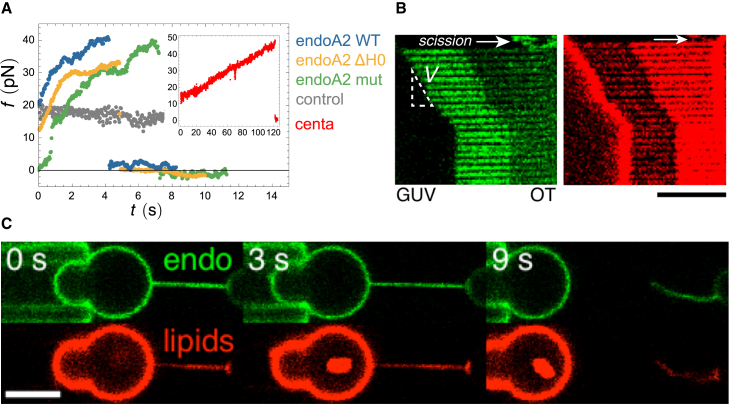
External Pulling Force Induces Scission of Endophilin-Scaffolded Tubes (A) Force *f*, as a function of time *t*, during the extension of a protein-scaffolded tube at speed *V*. For endoA2 WT, V=1.0 μm.s^−1^; for endoA2 ΔH0, V=1.15 μm.s^−1^; for endoA2 mut, V=0.95 μm.s^−1^; and for centaurin (centa), V=0.5 μm.s^−1^. Gray, control (no proteins), V= 0.3 μm.s^−1^ at vesicle tension $\sigma_{\text{v}}=$ 0.02 mN.m^−1^. (B) A kymogram of scission by extending a tube partially scaffolded by endoA2 (corresponding to the endoA2 WT force data in A), demonstrating that the protein scaffold adjacent to the GUV moves as the GUV is pulled leftward by external force. Vertical axis is time; total time is 8.5 s. Arrows indicate the severed tube. (C) Snapshots of scission by pulling an endoA2-scaffolded tube at 0.7 μm.s^−1^, highlighting the presence of endoA2 on the GUV. Scale bar, 5 μm. See also Figures S1, S2, and S3 and Movies S1, S2, and S3.

**Figure S2:**
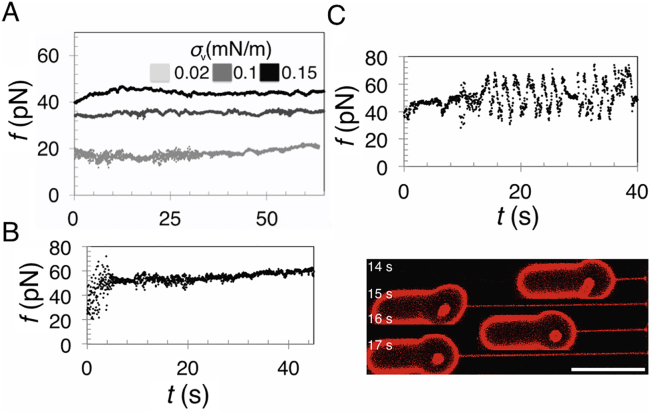
Elongating Bare Tubes Does Not Induce Scission, Related to Figure 1 (A) Tube pulling force, *f*, as a function of time, *t*, upon elongation of a tube at a rate of 0.3 μm.s^−1^, at different GUV tensions, *σ*_v_. (B) A control example with faster elongation: 1.3 μm.s^−1^ at 0.08 mN.m^−1^. (C) Aggressive elongation pulses of ∼20 μm.s^−1^ at 0.08 mN.m^−1^, where the vesicle was repeatedly brought back-and-forth. The force fluctuates due to pulses, as the lipids cannot equilibrate so fast, but rapidly equilibrates when ceasing to pull. The tube did not break after 12 pulses. Time stamp in fluorescent images corresponds to the time in the plot above. Scale bar, 10 μm.

**Figure S3:**
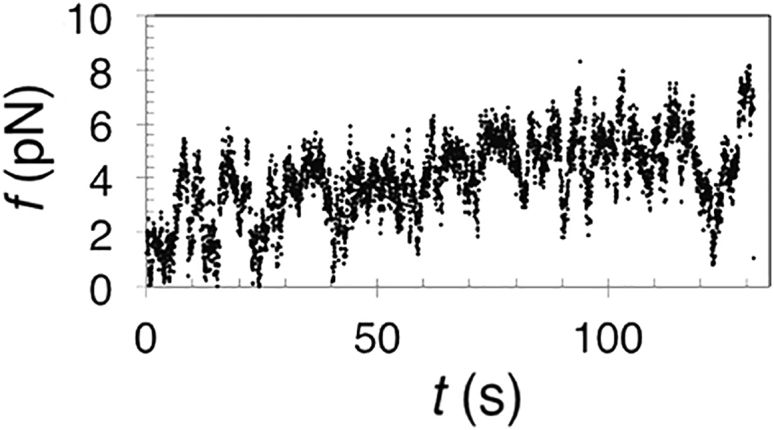
Very Slow Elongation Does Not Induce Scission, Related to Figure 1 Pulling an endoA2-scaffolded tube at 20 nm.s^−1^.

Nevertheless, there is evidence that endoA2-coated tubes can be cut upon elongation (Renard et al., 2015). Elongation has little effect on the force and stability of a bare membrane tube when pulled at biologically relevant speeds (Evans and Yeung, 1994); however, the presence of a protein scaffold might have a significant mechanical effect, potentially even to destabilize the tube. As a control, we elongated protein-free tubes at speeds up to 20 μm.s^−1^ and observed no scission. We measured only modest elongation-dependent force changes (Figure S2), consistent with Evans and Yeung (1994). We then elongated protein-scaffolded membrane tubes. We injected endoA2 near the pulled tube, which nucleates at the tube base and forms a scaffold, either partially or along the whole length of the tube (Figure 1; Figure S1) (Simunovic et al., 2016). Then, we extended the tube at constant speed *V* by displacing the aspirated GUV away from the optical trap. The tube-pulling force increased significantly upon tube extension until it dropped suddenly to zero, suggesting scission (Figure 1A). Scission was clearly observed by time-lapse confocal imaging of lipid and protein fluorescence (Figures 1B and 1C). In the case of endoA2, scission took place in 93% of the experiments (n = 43) at *V* = 50–8,000 nm.s^−1^ (Figure 1; Movies S2 and S3). Elongation at 20 nm.s^−1^ resulted in a very slow increase in *f* with no scission in the 150 s of the experiment (Figure S3).

**Figure S4:**
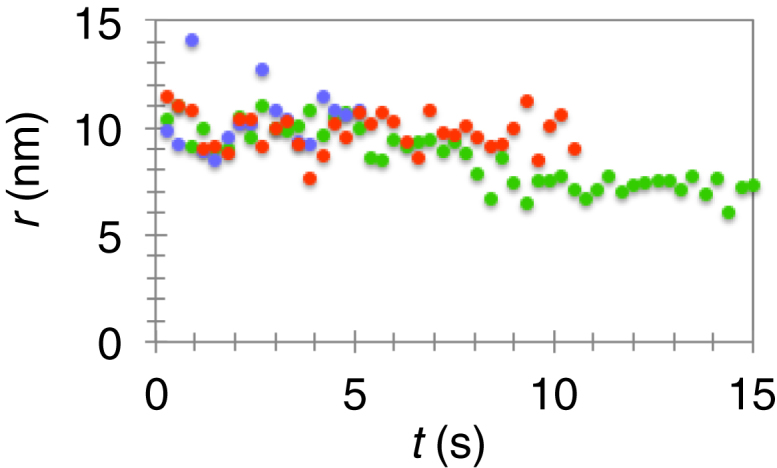
Measuring Scaffolded Tube Radius *r* versus Time *t* during Extension Leading to FDS, Related to Figure 1 Note, due to high-frequency imaging, some bleaching is observed. Different colors represent independent experiments at different pulling speeds: 5.1 μm.s^−1^ (purple), 0.17 μm.s^−1^ (orange), and 0.33 μm.s^−1^ (green).

Confocal imaging of partially scaffolded tubes demonstrated that during extension, the protein scaffold moved, together with the displaced GUV, away from the fixed bead, indicating that the scaffold is mechanically connected to the vesicle (Figure 1B). It also showed that the scaffolded tube radius remained unchanged under pulling (Figure S4). The relative movement between the scaffold and the membrane tube will be important in building a theoretical model and explaining the mechanism of membrane scission.

To see whether extension-driven tube scission is specific to endoA2-scaffolded tubes and the importance of AHs in this process, we considered centaurin and two endophilin mutants: one in which we truncated the N-terminal AHs (endoA2 ΔH0) and one in which we mutated a glutamate and an aspartate from the membrane-binding region of the N-BAR domain into lysines (E37K and D41K) (endoA2 mut). This reversal of charge enhances the binding strength of the BAR domain backbone to the membrane. Both mutants assemble into scaffolds on tubes, as we have shown (Simunovic et al., 2016). Although it has been shown that helix deletion does not impair the protein’s curvature-generating ability (Chen et al., 2016), scaffold formation requires seven times higher bulk protein concentration (Simunovic et al., 2016). We observed scission in all experiments for endoA2 ΔH0 (n = 6), in agreement with Renard et al. (2015), and in 92% of experiments for endoA2 mut (n = 13; in the only negative case, the bead was ejected from the trap). Finally, we observed five scission events upon elongation of centaurin-scaffolded tubes (n = 8; in the three negative cases, the bead was ejected) (Table S1). In conclusion, BAR proteins do not cut static membrane tubes; rather, they cut dynamically extended tubes via a mechanism that is not specific to the BAR protein backbone or the presence of AHs.

### Endophilin Scaffold Forms a Lipid Mobility Barrier

**Figure 2:**
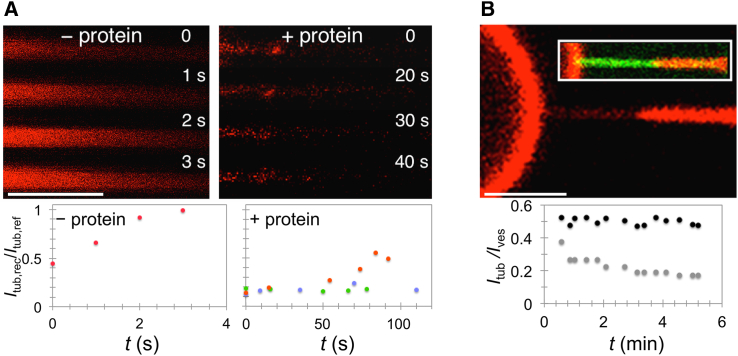
Protein Scaffold Forms a Lipid Diffusion Barrier (A) Confocal images after bleaching the bare tube (top left) and the protein-scaffolded tube (top right). Bottom: fluorescence intensity recovery of lipids in the scaffolded tube (*I*_tub,rec_) normalized by the prebleaching value (*I*_tub,ref_) as a function of time *t* of photo recovery. Plot colors represent different independent experiments. Scale bar, 3 μm. (B) Fluorescent image of a tube with coexisting scaffolded (thinner) and nonscaffolded (thicker) domains. Inset shows overlaid green (N-BAR domain) and red (lipid) channels. Graph shows the tube fluorescent intensity normalized by the vesicle intensity (*I*_tub_/*I*_ves_) on the nonscaffolded domain (black dots) and the calculation of what the tube radius would have been according to $r=\sqrt{\kappa /2\;\sigma_{\text{v}}}$ if tube tension were equilibrated (gray dots). During the experiment, $\sigma_{\text{v}}$ was increased stepwise. The scaffold creates a diffusion barrier preventing a quick reduction in *r* of the unscaffolded part. Scale bar, 3 μm.

Our observations that the force increases when scaffolded tubes are extended suggest a role for friction between the scaffold and the underlying tube in scission. In a different context, a force increase upon pulling tubes with transmembrane proteins or contaminants has been attributed to an augmented friction between the bilayer leaflets (Callan-Jones et al., 2016, Campillo et al., 2013). This scaffold-tube friction is expected to reduce lipid diffusion in the tube. To test this hypothesis, we monitored the fluorescence recovery after photobleaching (FRAP) of the entire tube. If the mobility of the bleached component is unperturbed, the fluorescence rapidly recovers due to the mixing of the bleached and the unbleached markers. As expected, the fluorescence recovery of protein-free tubes was fast (<5 s) (Figure 2A), consistent with the recovery time for free lipids in a tube (Berk et al., 1992). Conversely, in the presence of a scaffold formed by the N-BAR domain of endoA2, there was essentially no recovery, even after 90 s (Figure 2A). The weak decrease of the lipid diffusion coefficient on protein-free tubes with decreasing tube radius (Domanov et al., 2011) cannot account for this dramatic change. This reduction in lipid mobility is consistent with high friction between the tubular lipids and the surrounding protein scaffold (Merkel et al., 1989).

Another way to detect the influence of the scaffold on lipid mobility is by measuring the change in *r* resulting from a change in vesicle tension $\sigma_{\text{v}}$. For a protein-free tube, *r* adjusts within seconds after a $\sigma_{\text{v}}$ change (Dommersnes et al., 2005). We considered a partially scaffolded tube, in which the scaffold was located between the protein-poor part of the tube and the GUV. When the vesicle tension was increased stepwise, with a waiting period of about a minute between steps, there was no detectable change in the protein-free *r*, as measured by lipid fluorescence (Figure 2B). This observation suggests that the tension in the protein-free tube was not equilibrated with the vesicle, likely because of the friction between the scaffold and the tube lipids.

### Modeling Friction between a Protein Scaffold and a Membrane Tube

Our measurements of the increasing force as scaffolded tubes are extended and the reduction in lipid mobility as detected by FRAP suggest that friction opposes the relative movement between the scaffold and the underlying membrane. Here, we model this hypothesis and test it against our force measurements.

**Figure 3:**
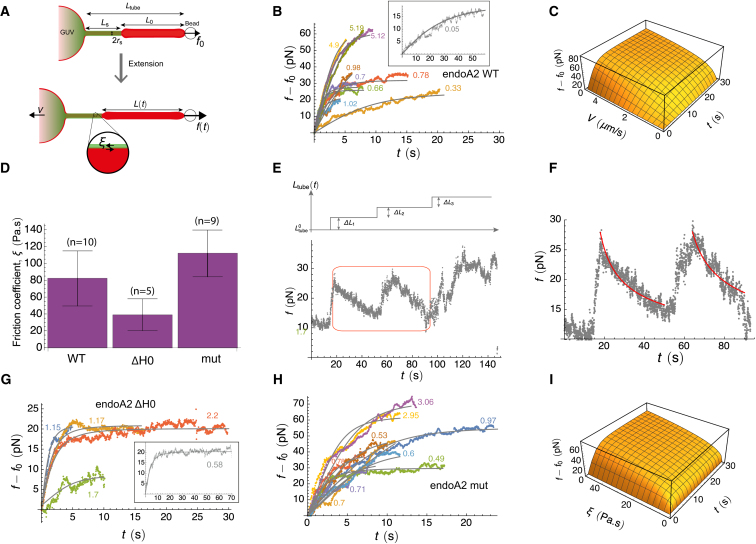
Extending Endophilin-Coated Tubes Leads to Force Increase (A) Illustration of a protein-scaffolded tube extended at speed *V*. Relative motion between scaffold (green) and membrane tube (red) results in friction, with coefficient ξ, and an increase in force *f*. (B) Force versus time for WT endophilin-coated tubes, pulled at a constant speed *V*, for ten vesicles. Each dataset is fitted with Equation 3 for the given value *V* (as labeled, given in micrometers per second). For clarity, the initial force $f_{0}$ has been subtracted. Inset: scission of a vesicle pulled at the lowest speed occurs on a longer timescale and is shown separately. (C) 3D plot of f versus t and *V*, calculated from Equation 3, showing the increase of force with pulling speed, all other parameters remaining constant ($f_{0}$ = 2 pN, $L_{0}$ = 0.8 μm, and ξ = 30 Pa.s). (D) Bar graph comparing the mean friction coefficient ξ for endoA2 WT, endoA2 ΔH0, and endo mut (error bars indicate SEM). Average values are ξ=80± 30 Pa.s (endoA2 WT), ξ=39±19 Pa.s (endoA2 ΔH0), and ξ=112±27 Pa.s (endoA2 mut). (E) Force after a sudden increase in tube length. Relaxation of the force occurs following two length jumps, $\Delta L_{1}$ = 3 μm and $\Delta L_{2}=4$ μm. (F) Fits to the force relaxation after the two steps (red boxed region in H) yield ξ=35±0.8 Pa.s and ξ≈73±2 Pa.s (95% confidence limit [CL]). The total bare length was estimated as L=4.95 μm (obtained by integrating (d/dt)(L/f)=0 across the step) after the first step and L=8.6 μm after the second one. The relaxation data were fitted numerically by solving Equation 2. The bending stiffness of the membrane was taken to be $\kappa =45\;k_{\text{B}}T$ for all fits (Simunovic et al., 2016). (G) Force versus time endoA2 ΔH0-coated tubes, pulled at a constant speed (n = 5). Inset: scission of a vesicle pulled at the lowest speed occurs on a longer timescale and is shown separately. (H) Force versus time endoA2 mut-coated tubes, pulled at a constant speed (n = 9). (I) 3D plot of f versus t and ξ, calculated from Equation 3, showing the increase of force with friction, all other parameters remaining constant (*V* = 2 μm.s^−1^, $f_{0}$ = 4 pN $L_{0}$ = 0.8 μm). See also Figure S4.

When protein-scaffolded tubes were extended at constant velocity *V* (schematized in Figure 3A), we found that the force f(t) increased at short times after extension began and then tended toward a constant value (Figures 1A and 3B). The saturating force $f_{\infty }$ increased with *V* (Figure 3B). These observations suggest a viscoelastic-like response: at short times, the behavior is elastic, because lipid flow from the vesicle to the tube is impeded by friction, and *f* increases due to the increased bending energy of the tubular membrane. At longer times, a balance between tube extension and lipid influx underneath the scaffold sets in, the force becomes constant, and friction dominates.

**Figure S5:**
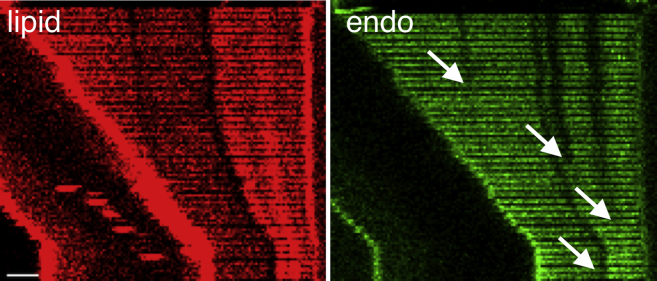
Scaffold Breakup upon Elongation of a Fully Covered Tube, Related to Figure 3 Kymogram of a tube fully covered by a scaffold of N-BAR domain of endoA2, showing that the scaffold breaks apart upon elongation. This implies that fully and partially scaffolded tubes are equivalent, as far as FDS is concerned. Arrows point to scaffold breaks observable by our microscope. Scale bar, 2 μm.

We consider at time *t* a tube of length $L_{\text{tube}}\left(t\right)\;$ coated with a protein scaffold of fixed radius $r_{\text{s}}$. Because tubes were often found to be incompletely coated (Figure 1B), the total length is written $L_{\text{tube}}\left(t\right)=L_{\text{s}}+L\left(t\right)$, where $L_{\text{s}}$ and L(t) are the lengths of scaffolded and unscaffolded tubes (Figure 3A). The unscaffolded tube is expected to be cylindrical, with radius r(t), at distances of the order r away from the scaffold interface (Morlot et al., 2012). In cases in which tubes appeared to be initially fully covered, we often observed that almost immediately after extension began, gaps in the scaffold appeared (Figure S5). This effectively renders the tubes incompletely coated for most of the extension period, and our hypothesis of partially coated tubes is generally valid. Upon extension, we assume, in agreement with experimental observations, that the scaffold is rigid and does not change its length (Figure 1C); therefore, $L\left(t\right)=L_{0}+\text{Δ}L\left(t\right)$, with $L_{0}$ as the initial length of uncoated tube and ΔL(t) as the controlled change in tube length. For constant speed extension, ΔL(t)=Vt, and for a sudden step, $\text{Δ}L\left(t\right)=\text{Δ}L_{\text{step}}$.

A key ingredient in the model is that elongation of a scaffolded tube causes an increase in tube tension, an effect that does not occur for bare membrane tethers (Derényi et al., 2002). Adapting a model of tether pulling from cytoskeleton-attached membranes (Brochard-Wyart et al., 2006), friction dynamically relates the tube tension σ(t) to the speed $v_{\text{l}}$ of lipids underneath the scaffold:

(Equation 1) $$\sigma \left(t\right)=\sigma_{0}+\xi \;v_{\text{l}},$$

where $\sigma_{0}$ is the tension before extension begins and ξ is the scaffold-membrane friction coefficient. This friction reflects dissipation due to relative movement between membrane lipids and proteins forming the scaffold (Figure 3A). Equation 1 is an integrated expression of the linear momentum conservation law in the lipid layer (see Supplemental Information for a detailed discussion).

The increase in tension due to friction leads to a change in the tube pulling force, which can be measured directly. Lipid membranes are practically incompressible (Rawicz et al., 2000), which implies $d\left(rL\right)/dt=r_{\text{s}}v_{\text{l}}$, and using known relations among r, *f,* and σ for the unscaffolded tube (Derényi et al., 2002), it can be shown (see Supplemental Information) that Equation 1 leads to

(Equation 2) $$\frac{d}{dt}\left(\frac{L}{f}\right)=\frac{f^{2}-f_{0}^{2}}{16\pi^{3}\kappa^{2}\xi /r_{\text{s}}}.$$

This equation can be solved for f(t) for the experimental protocols for L(t).

By applying our model to f(t) for endoA2-scaffolded tubes, we were able to estimate the friction coefficient ξ. Moreover, because endoA2 mutants interact differently with the membrane than does endoA2 wild-type (WT), we expect that ξ reflects these differences. Solving Equation 2 for $L\left(t\right)=L_{0}+Vt$ yields an explicit expression for f(t):

(Equation 3) $$f\left(t\right)=f_{0}\frac{1+Vt/L_{0}}{{\left(1+\frac{{\left(1+Vt/L_{0}\right)}^{3}}{16\pi^{3}\kappa^{2}\xi V/\left(r_{\text{s}}f_{0}^{3}\right)}\right)}^{1/3}};$$

see Figure 3C for the variation of *f* with *t*. The preceding equation is valid as long as $V\gg r_{\text{s}}f_{0}^{3}/\left(16\;\pi^{3}\kappa^{2}\xi \right)$; see Supplemental Information. In practice, as discussed later, this equality requires that V≳50 nm.s^−1^, which is always the case.

Equation 3 recapitulates the viscoelastic-like properties announced earlier, revealing two distinct regimes. At times that are short compared with

(Equation 4) $$t^{*}={\left(\frac{16\pi^{3}\kappa^{2}\xi L_{0}^{3}}{r_{\text{s}}f_{0}^{3}\;V^{2}}\right)}^{1/3},$$

the tube is elongated elastically at a fixed number of lipids and the force increases linearly with t with slope $f_{0}V/L_{0}$. For times greater than $t^{*}$, lipid influx across the scaffold occurs and the force saturates to

(Equation 5) $$f_{\infty }\approx {\left(\frac{16\pi^{3}\kappa^{2}\xi }{r_{\text{s}}}\right)}^{1/3}V^{1/3}.$$

In addition, the tension in the tube builds to $\sigma_{\infty }=f_{\infty }^{2}/\left(8\pi \kappa \right)\propto {\left(\xi V\right)}^{2/3}$. Thus, our proposed scission mechanism via tension-caused membrane pore nucleation (discussed later) is seen to depend crucially on friction and pulling speed.

Our model allows us to quantitatively determine the effects of protein scaffolding on tube dynamics. The *f* versus *t* data for endoA2 WT-scaffolded tubes were first obtained by performing several elongation experiments (n = 10) (Figure 3B). For each experiment, the pulling speed *V* was held constant; *V* ranged from 50 nm.s^−1^ to >5 μm.s^−1^. Fitting these datasets with Equation 3 allowed us to determine an average friction coefficient ξ (Figure 3D). From the data, we note that with increasing *V*, *f* rose faster and, at a long time, saturated at higher $f_{\infty }$ values, in agreement with the model prediction (Figure 3C).

As an independent test of our model, we performed a force relaxation experiment on a WT-scaffolded tube, in which the length of the tube was increased stepwise and the subsequent force behavior was monitored (Figure 3E). Fitting the force relaxation after two steps (Figure 3F) by solving Equation 2 (see Supplemental Information for details) yields good agreement with the constant speed elongation experiments. Thus, the friction between a protein scaffold and a membrane tube is a general mechanical property of dynamic tubes. It provides a quantitative measure of the scaffold’s ability to create a local tension increase on the tube, which as we show, is a prerequisite for scission.

Validation of the model was found by comparing the ways in which endoA2 WT and two mutants, endoA2 ΔH0 and endoA2 mut, affect the tube force. For comparable elongation speeds, *f* attains a lower saturation force for endoA2 ΔH0 (Figure 3G) than for WT (Figure 3B), whereas *f* tends to a slightly higher value for endoA2 mut (Figure 3H). Based on our theoretical model (Figure 3I), these trends suggest that the friction between an endoA2 ΔH0 scaffold and the lipid tube is lower than for WT and greater for endoA2 mut scaffold, confirmed by fitting the data for the two mutants (Figure 3D). This is consistent with the effect of insertion of AHs shown in Ambroso et al. (2014) and suggests a correlation between binding affinity and friction. As we show now, this scaffold friction-generated force increase provides a natural mechanism to cut tubes, which we refer to as FDS. Box 1 presents a summary of the FDS mechanism.

Box 1 **Physics of FDS**

Consider a membrane tube that is partially coated by a BAR protein scaffold. The scaffold imposes a frictional force on the underlying tube. When the tube is elongated at constant speed *V*, lipid flow underneath is very slow due to friction and cannot easily pass into the protein-free tube. The protein-free tube therefore becomes thinner, increasing the bending energy of the membrane. Eventually, a steady state is reached in which the tube radius no longer changes and the force, *f*, reaches a constant value that varies with *V* and the friction coefficient *ξ*.

**Figure:**
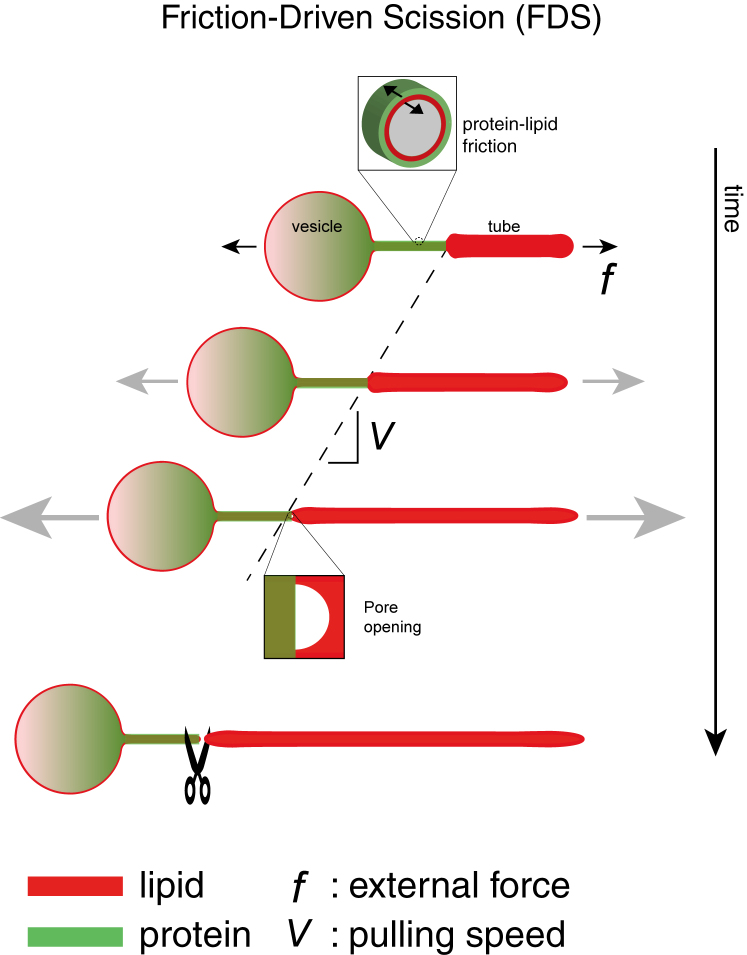

As *f* increases, so does the membrane tension σ along the bare part of the tube, increasing the probability of a pore in the membrane leading to scission. Our model predicts that faster pulling induces FDS at a higher breaking force $f_{\text{break}}$ but at a shorter time $t_{\text{break}}$, which we experimentally verified. Tubes scaffolded by mutated proteins whose ξ is lowered break at longer times but at lower forces than WT at comparable pulling speeds. In cells, this pulling force is likely provided by molecular motors.

### Nucleation of Pores in the Membrane Causes Rupture of Membrane Tubes through FDS

Three routes to scission of membrane tubes can be considered. First, local tube pinching from a radius $r_{0}$ down to $r_{\text{i}}\approx 3$ nm leads to scission via a hemifission intermediate state (Kozlovsky and Kozlov, 2003). Second, it has been proposed that line tension, which arises at the boundary between lipid domains and acts to reduce the boundary length, could constrict tubes enough to cause scission (Allain et al., 2004, Römer et al., 2010). The characteristic scission times of an endoA2-scaffolded tube by each of these mechanisms can be estimated and are orders of magnitude larger than what we have measured; see Supplemental Information for details. In the last route, scission is preceded by the nucleation and growth of a pore in membrane at lysis tension, a process that has been studied extensively in synthetic membrane systems (Evans et al., 2003). In our case, we have shown that scaffold friction leads to a force increase and thus to a tension increase in the bare membrane tube upon elongation. Assuming a tube extension rate of 1 μm.s^−1^, the tension increases roughly at 0.1 mN.m^−1^.s^−1^; according to Evans et al. (2003), this loading rate corresponds to a lysis tension of about 1 mN.m^−1^ and a tube lysis force of about 100 pN. This value is on the order of magnitude of the force attained in our extension experiments (Figure 3), indicating that scission through membrane lysis is a plausible mechanism of FDS.

**Figure 4:**
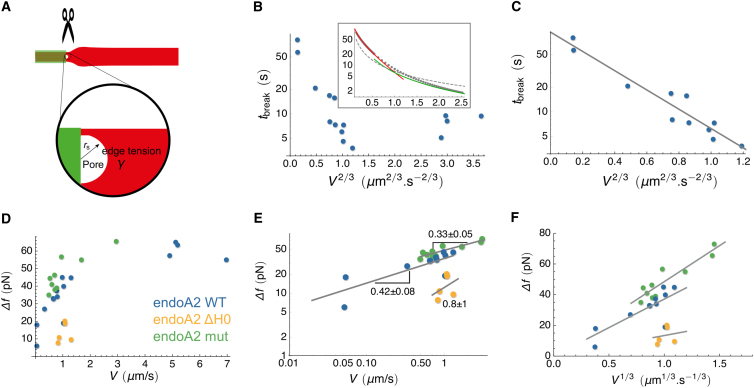
Tube Scission Time and Force as a Function of Pulling Speed (A) Illustration of FDS via nucleation of a pore in the membrane at the scaffold and membrane edge. Opening a pore releases bending and stretching energy, while creation of an exposed edge costs energy per unit length, given by the line tension γ. See also Figure S5 and Movie S3. (B) Scission time data versus pulling speed *V* for endoA2 WT. Data ($\text{log}\left(t_{\text{break}}\right)$ versus *V*^2/3^) suggest two speed regimes. Inset: numerical calculation of $\text{log}\left(t_{\text{break}}\right)$ versus *V*^2/3^ (round symbols) for *f* given by Equation 5 (see Supplemental Information). Asymptotic expressions for $t_{\text{break}}$ (red line: $t_{\text{break}}\gg t^{*}$, see Equation 6; green line: $t_{\text{break}}\ll t^{*}$, see Equation S22) confirm that two pulling speed regimes exist. The crossover time $t^{*}\left(V^{2/3}\right)$ is shown by a dashed line. (C) Scission time data and fit at low *V* for endoA2 WT. A linear fit, using Equation 6, yields ξ=56±16 Pa.s. Error reflects the SE in the fit parameter obtained using a nonlinear fitting method. (D) Force supplied by tube extension until scission Δf versus *V*. Data are shown for endoA2 WT and mutants. (E) Log-log plots of Δf versus *V* at low *V* is consistent with the theory prediction that $\Delta f∼V^{1/3}$. (F) Fits to Δf versus *V*^1/3^ at low *V* yield, using Equation 5, ξ=30±12 Pa.s (endoA2 WT), ξ=1.4±2 Pa.s (endoA2 ΔH0), and ξ=66±6 Pa.s (endoA2 mut). Error reflects the SE in the fit parameter obtained using a nonlinear fitting method.

To test our hypothesis of FDS, we now investigate in detail how the scission force and rupture time depend on the extension rate, as illustrated in Figure 4A. When extended at constant speed *V*, endoA2-coated tubes broke at a time $t_{\text{break}}$ that was found to decrease with increasing *V* (Figure 4B). Furthermore, tube breaking appears to be a stochastic process. These combined observations suggest that tube scission involves thermal activation over a barrier that is lowered by the applied force.

To model this effect, we assume heterogeneous membrane pore nucleation at a scaffold edge—along the tube, at the GUV neck, or at the tube end—because most tubes were found to break at these locations (Movies S2 and S3). Nucleation of a roughly semicircular-shaped pore of radius $r_{\text{s}}$ is energetically opposed by forming a free membrane edge, with edge tension γ, but is favored by releasing bending and stretching energy (Figure 4A). As can be shown (see Supplemental Information), these considerations lead to an energy barrier $W\left(t\right)≃\pi \;\gamma r_{\text{s}}-r_{\text{s}}^{2}\;f{\left(t\right)}^{2}/8\pi \kappa$, which is elongation speed dependent. The probability P(t) for nucleation of a pore of size $r_{\text{s}}$ at time *t* after elongation begins can then be related to W using Kramers’s theory for thermally activated escape (Kramers, 1940); see Supplemental Information for details. Following Evans et al. (2003), the scission time $t_{\text{break}}$ is identified with the peak of P(t). This leads to analytical expressions for $t_{\text{break}}$ in the limits $t_{\text{break}}\gg t^{*}$ and $t_{\text{break}}\ll t^{*}$, i.e., for slow and fast pulling; see the inset of Figure 4B, in which the crossover between the regimes is found to occur for V∼ 1 μm.s^−1^. In the limit $t_{\text{break}}\gg t^{*}$, we obtain

(6) $$t_{\text{break}}≃\tau \;\text{exp}\left[-\frac{\pi }{k_{\text{B}}T}{\left(\frac{\kappa \xi^{2}r_{\text{s}}^{4}\;V^{2}}{128}\right)}^{1/3}\right],$$

where τ depends algebraically on *V*; thus, the dependence of $t_{\text{break}}$ on *V* is dominated by the exponential. Representing the endoA2 WT data as ln$\left(t_{\text{break}}\right)$ versus $V^{2/3}$ reveals the two pulling regimes (Figure 4B). By performing a linear fit at low pulling speeds, we obtain another determination of the friction coefficient (Figure 4C), in good agreement with earlier values (Table S2). This result strongly supports our model of FDS through pore nucleation.

The dependence of the tube force at scission, $f_{\text{break}}=f\left(t_{\text{break}}\right)$, on pulling speed provides a second test of FDS. For $t_{\text{break}}\gg t^{*}$, the force is essentially saturated; therefore, according to Equation 5, the extra force provided by tube elongation until scission is Δf≡ $f_{\text{break}}-f_{0}∼{\left(16\pi^{3}\kappa^{2}\xi /r_{\text{s}}\right)}^{1/3}V^{1/3}$. This prediction is borne out well by our data (Figures 4D and 4E). In addition, a fit of Δf versus $V^{1/3}$ at low V was done to obtain the friction coefficients for endoA2 WT, endoA2 ΔH0, and endoA2 mut (Figure 4F). We see that two separate analyses—fitting f(t) and $f_{\text{break}}$—show the same effect of mutation on the friction coefficient, confirming the validity of the scission model; see Table S2 and further discussion in the Supplemental Information on the different determinations of ξ.

### Molecular Motors and BAR Proteins Induce Scission

Tube elongation in cells is often mediated by molecular motors. Motors walking on microtubules in vitro can extract membrane tubes at speeds ranging from a few tens to hundreds of nanometers per second (Leduc et al., 2004). Higher speeds, close to 1 μm.s^−1^, have been observed in vivo (Sciaky et al., 1997, Skjeldal et al., 2012), exceeding the pulling speed of ∼50 nm.s^−1^ that we found was needed for FDS. Considering that dynein was shown to elongate Shiga or Cholera toxin-containing tubular membranes under low-ATP conditions in CIE (Day et al., 2015, Renard et al., 2015), we tested whether this motor can pull tubes quickly enough to trigger FDS.

**Figure 5:**
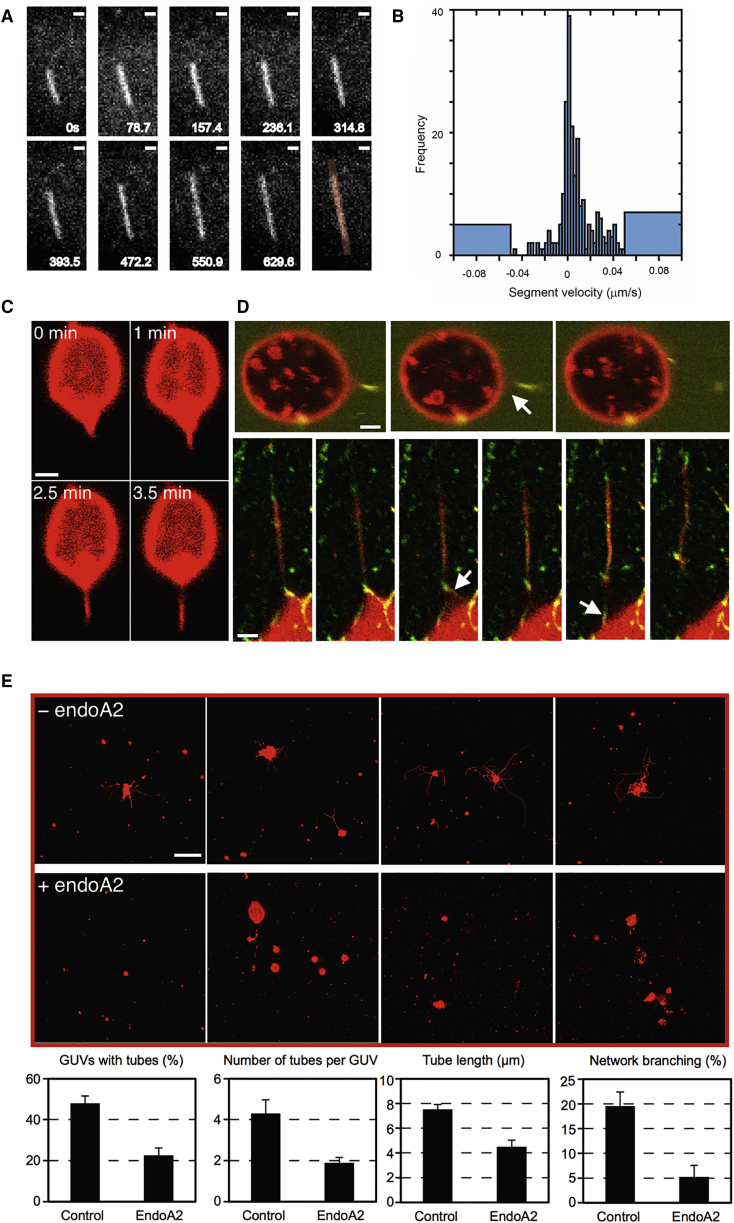
Scission of Tubes Pulled by Microtubule-Associated Motors (A) Time series showing the formation and motion of CTxB containing tubular invagination in an ATP-depleted COS-7 cell. Scale bar, 1 μm. (B) Distribution of tube-tip velocities measured from a kymogram at each time segment (225 total segments) from a total of 25 tubes. (C) Pulling a tube from a GUV by kinesin in the absence of other membrane-curving proteins. The onset of tube pulling is minutes after introducing ATP into the system. Fluorescence, lipids. Scale bar, 2 μm. (D) Time lapse of two experiments showing scission of endophilin-coated tubes mediated by kinesin. Arrows point to observed scission locations just before breakage. The second example shows two breakage events of a branched tube. Green, endophilin; red, lipids. Scale bars, 2 μm. (E) Steady-state observations of membrane scission mediated by kinesin and endophilin. Shown are representative images of vesicles in the presence of kinesin and ATP without endophilin A2 (−endoA2) and in the presence of 2.5 μM endophilin A2 (+endoA2). Fluorescence, lipids. Scale bar, 20 μm. Plots show quantification of the frequency and morphological characteristics of tubes in the control (kinesin + ATP) and in the presence of endoA2 (kinesin + ATP + endoA2). Data are mean ± SEM. Observations taken after 30 min of reaction time. Measurements taken from three independent experiments with a total of 163 GUVs (−endoA2) and 143 GUVs (+endoA2). See also Figure S6 and Movies S4 and S5.

**Figure S6:**
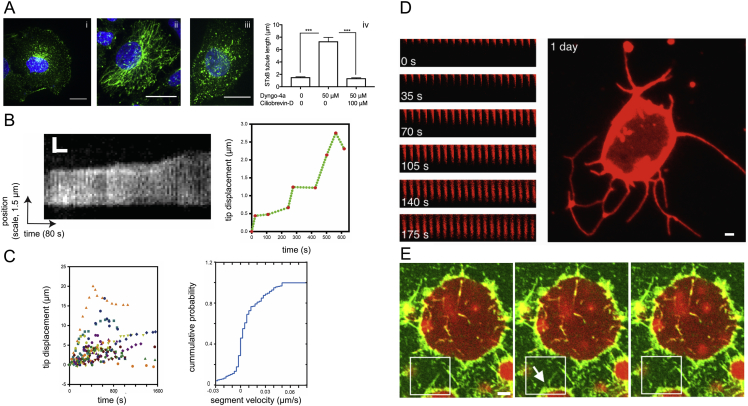
Molecular Motors Work with EndoA2 to Induce FDS, Related to Figure 5 (A) Effect of dynein motor on STxB tubules. Incubation of HeLa cells for 1 hr at 37°C with (i) untreated (control), (ii) 50 μM dyngo-4a (dynamin inhibitor), and (iii) 50 μM dyngo-4a +100 μM ciliobrevin-D (dynein inhibitor), followed by incubation with STxB-A488 (5 μg.mL^−1^) for 10 min. Determination of tube length on fixed cells; ^∗∗∗^p < 0.001 (One-way Anova test). Blue: Hoechst dye; green: STxB-A488. Data are mean ± SEM of two independent experiments (n = 25 cells per condition). (B) Left: kymogram of a CTxB-containing tube elongated by dynein in an ATP depleted COS-7 cell. The position of the line used to generate the kymogram is highlighted in orange in Figure 5A in the main text. Cholera toxin B-subunit is fluorescently labeled. Right: the corresponding trajectory of the tubule tip as a function of time as traced from the kymogram. (C) Left: traces of tip displacement over time for 25 different tubules. Each individual tubule is indicated by a different symbol. Right: the cumulative probability plot of the resulting 228 measurements of segment velocity calculated as the slope of the lines between individual data points. Note, the corresponding histogram is in Figure 5A in the main text. (D) Left: kymogram of a tubule extruded from a GUV by kinesin motors (split into six lines, with each line indicating the time of the first image), showing the growth of the tubule shown in Figure 5C in the main text. The total vertical length in each segment is 3.55 μm. Right: another example with many extruded tubules, taken one day following the experiment. The chamber was sealed with putty sealant and kept in the fridge overnight. Lipids are fluorescent. Scale bar, 2 μm. (E) Time-lapse microscopy of FDS of endoA2-covered tubules pulled by kinesin motors. The arrow points to a scission location just prior to breakage. Overlaid fluorescence of lipids (red) and endoA2 (green). Scale bar, 2 μm.

First, we confirmed that dynein drives the elongation of tubes containing Shiga toxin subunit B (STxB) in CIE under normal ATP levels in cells (see Supplemental Information) (Figure S6A). Then, to measure the tube-pulling rates in vivo, we tracked the motion of tubes containing Cholera toxin subunit B (CTxB) on live-cell imaging data published in Day et al. (2015). Kinematic analysis (see STAR Methods) revealed that the extension speed of dynein-pulled tubes in low-ATP conditions was <50 nm.s^−1^, with the longest tubes extending <20 nm.s^−1^ (only 7 of 228 time segments from a total of 25 tubules reached speeds > 50 nm.s^−1^ and never >90 nm.s^−1^) (Figures 5A and 5B; Figure S6B). Two observations can be made: (1) under normal ATP conditions, motors most likely exceed the threshold velocity of 50 nm.s^−1^ and (2) not observing tubes pulled at high speeds may indicate they have been cut.

To directly observe FDS by motor proteins, we devised a biomimetic system combining GUVs, endoA2, and kinesin motors. Kinesins were previously shown to pull out tubes from GUVs in vitro (Koster et al., 2003, Leduc et al., 2004, Roux et al., 2002). We first confirmed that kinesin successfully extracted tubes from GUVs composed of lipids used in this study (see STAR Methods) (Figure 5C; Figure S6D). Next, during tube extension by motors, we injected endoA2 into the system, which quickly became enriched on motor-pulled tubes. Seconds later, we observed scission (four observations in three experiments) (Figure 5D; Figure S6E; Movies S4 and S5). We quantified these scission events by comparing the prevalence of motor-pulled tubes from GUVs in the presence and absence of endoA2 at 30 min postincubation (Figure 5E). In the presence of endoA2, the number of GUVs with long tubes, as well as the number of tubes per GUV, decreased more than 2-fold. Furthermore, motor-pulled tubes were almost twice as short in the presence of endoA2 (Figure 5E). This confirms that motors work with endoA2 to induce scission. EndoA2 alone induces tubulation of GUVs (Figure S1A); therefore, observing fewer tubes in the presence of endoA2 and motors strengthens the conclusion that their pulling velocity is sufficient to induce scission. We also observed fewer branches, likely indicating that tubes broke from networks and not just from the GUVs.

To summarize, molecular motors extending tubes at speeds of a few tens to hundreds of nanometers per second can provide the force needed to cause scission. Under physiological conditions, it is expected that higher tube extension speeds are achievable; thus, motor-aided scission events are even more prevalent.

## Discussion

Scission of membrane invaginations is an essential component of endocytosis and intracellular trafficking. Although several membrane-bound trafficking factors have been identified in scission (e.g., dynamin, endophilin, and actin), a global, mechanistic understanding of how they function has remained elusive, with the notable exception of dynamin-driven neck constriction. FDS is a generic mechanism and requires that the tube-bound proteins impose strong friction on the underlying membrane. It also requires two mechanical conditions: (1) an external force elongating the tube and (2) an anchoring of the protein structure at the tube base. In the case of CIE mediated by endophilin, as studied here, a scaffold of endophilin BAR domains imposes the frictional force on the membrane tube, mechanically connected to the neck, while the motor dynein provides the pulling force for tube extension, enabling FDS.

### Scaffold Slows Lipid Diffusion

Friction results from interactions between the protein scaffold and the lipid membrane. We found that the scaffold dramatically reduces the mobility of lipids underneath, with important consequences for sorting in nascent endocytic membrane carriers. Once a scaffold is formed on a preendocytic bud, diffusion of membrane-containing cargoes on the bud back to the plasma membrane is impaired, which could thereby kinetically trap them. This effect may, however, be opposed by the influx of new cargoes across the scaffold by advection due to tube elongation. Advection would occur on timescales greater than the characteristic time $t^{*}$ set by the friction coefficient and the pulling speed (see Equation 4). As a case in point, microcompartmentation of certain lipids is critical for auxilin recruitment to the clathrin-coated bud just before scission, enabling the uncoating of the fully formed clathrin-coated vesicle (Massol et al., 2006). This was suggested to be due to diffusion-limited accumulation of auxilin-binding lipids in the bud at the right time and place upon scaffolding by BAR domain proteins.

### Scission of Spherical versus Tubular Vesicles

A surprising finding of our work is that endoA2 forms a scaffold that stabilizes preformed membrane tubes, whereas the same protein spontaneously fragments small spherical vesicles (Boucrot et al., 2012). What is the origin of this apparent discrepancy? The initial morphology of the membrane could be crucial in determining whether the insertion leads to membrane scission or stabilization of the curvature. Earlier studies indicate that there is a fundamental difference in the way BAR proteins interact with spherical versus cylindrical membranes (Ambroso et al., 2014). In fact, α helices favor positive Gaussian membrane curvature and are thus able to drive a topological transformation from a vesicle to several smaller ones (Boucrot et al., 2012). This effect depends on the depth of insertion: as the insertion gets closer to the bilayer middle plane, the effect becomes smaller (Campelo et al., 2008). On small spherical vesicles, the BAR domain backbone is not tightly bound to the membrane and the insertion is therefore shallow (Ambroso et al., 2014), favoring vesiculation. In contrast, on a preformed nanotube, the same proteins bind closely to the tube, forming a scaffold (Simunovic et al., 2016); AH inserts deeper (Ambroso et al., 2014), and there is no incentive to spontaneously undergo scission. This effect resolves the apparent discrepancy. The dueling effects of the insertion and the backbone can be illustrated by considering CME. In the late stage of CME in mammalian cells, the clathrin-coated spherical membrane bud is connected to the flat membrane by a short neck with negative Gaussian curvature. In this case, insertion of α helices—from endophilin or from the epsin N-terminal homology (ENTH) domain of epsin—destabilizes the neck, favoring scission. The shape of the neck prevents its stabilization by the BAR backbone. In CIE or in our tube experiments, we expect the backbone effect to overwhelm the insertion one. However, in CME in yeast, which poorly relies on dynamin, the BAR domain of Rvs161/167p (homologous to mammalian amphiphysin 1) stabilizes the neck into a long cylinder; here, scission could occur through FDS, generated by the pulling force of actin.

We therefore propose that depending on the presence of α helices, BAR domain proteins function directly via two distinct mechanisms in scission. First, if the nascent bud is spherical, attached to the donor membrane (i.e., the plasma membrane for CMEs in mammalian cells or the endoplasmic reticulum and Golgi apparatus for coat protein II [COPII]- and coat protein I [COPI]-coated vesicles, respectively) by a short, curved neck, then its shape is inherently destabilized by insertions (those of Sar1 for COPII and of Arf1 for COPI). Second, if the bud is tubular, it is not vulnerable to scission via insertions but might undergo FDS. In this case, the donors are the plasma membrane (for endocytosis of toxins and growth factors such as epidermal growth factor [EGF]) and possibly endosomes, where sorting nexins provide scaffolding, and the elongation force comes from actin polymerization and/or motors, e.g., in Traer et al. (2007). Furthermore, it seems likely that yeast CME, and mammalian CME at high membrane tensions function in a hybrid manner: they combine a spherical bud with a long, stabilized neck and require actin to undergo scission.

### Role of the Cytoskeleton

**Figure 6:**
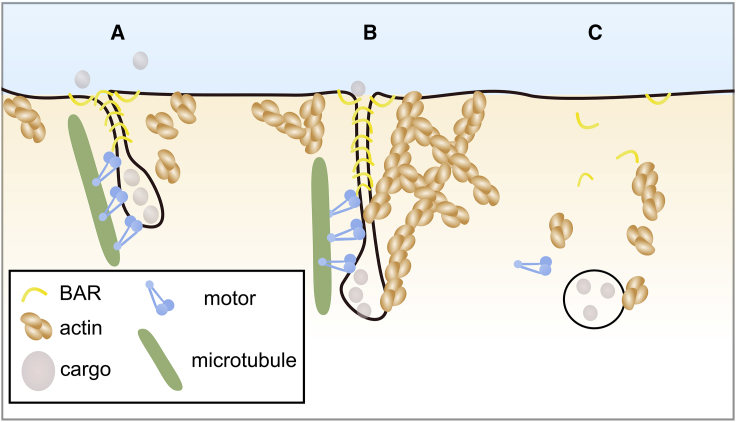
Schematics of Endocytosis by FDS Hypothetic role of FDS in vivo enabled by BAR domain scaffolding. (A) BAR domain proteins, such as endoA2, bind to the cytosolic leaflet of endocytic membrane invaginations, forming a scaffold. The membrane tube containing cargoes is extended by microtubule-based molecular motors, such as dyneins or kinesins. (B) Tube elongation is aided by actin polymerization, up to the point at which FDS occurs. (C) Eventually, the BAR domain proteins, the motors, and actin disassemble from the endocytosed vesicle, which continues along the endocytic pathway.

FDS is a generic scission module that hinges on a few basic elements, namely, a protein scaffold and a tube extension force; see Figure 6 and Box 1. Our discovery that molecular motors walking on microtubules can pull quickly enough to break a membrane tube when associated with protein scaffolds opens exciting new routes for future investigations. Depending on the microtubule orientation—i.e., at the plasma membrane or at the Golgi apparatus—different motor families are expected to be involved in tube scission. Alternatively, the pulling force for FDS could be provided by actin polymerization (Figure 6). Actin has been shown to be involved widely in endocytosis (Mooren et al., 2012), particularly in CME in mammalian and yeast cells (Boulant et al., 2011, Ferguson et al., 2009, Grassart et al., 2014) and in CIE (Renard et al., 2015).

### Possible Interplay with Dynamin

FDS provides a scission mechanism in situations in which dynamin is dispensable. Nevertheless, both mechanisms can coexist and be cumulative. In mammalian cells, CME generally involves dynamin for clathrin vesicle budding, although synergistic effects between N-BAR domain proteins and dynamin on their mutual recruitment and dynamin guanosine triphosphatase (GTPase) activity have been reported (Meinecke et al., 2013, Neumann and Schmid, 2013, Sundborger et al., 2011, Taylor et al., 2012, Yoshida et al., 2004). In CIE, endophilin-mediated FDS acts in parallel with dynamin GTPase-induced scission (Renard et al., 2015), thereby building robustness into the process. We propose here that N-BAR domain proteins can additionally assist dynamin-mediated scission (1) by establishing a mechanical docking for the dynamin coat to the plasma membrane, allowing FDS to occur for the dynamin-scaffolded neck if actin polymerization or motors provide extensional force, and (2) by directly contributing to neck scission by FDS.

### Fast Endocytosis

Endophilin A1 and A2 are known to participate in fast endocytosis in neurons, occurring on a timescale of seconds (Boucrot et al., 2015, Llobet et al., 2011). Our experiments revealed that endophilin-dependent FDS, enabled by microtubule-based motors, occurs on the order of a few tens of seconds. This difference in timescales could be related to other scission modules (dynamin and actin), which can act in parallel in vivo and make the scission process very fast. It could also be due to the relatively higher proportion of polyunsaturated lipids in neuronal membranes (Yang et al., 2012), as compared with our experiments. The presence of these lipids could result in a decrease of the membrane bending rigidity κ and an increase in the number of lipid packing defects (Pinot et al., 2014). Although the effect of a decrease in κ on the scission time is not expected to be strong (see Supplemental Information), the effect of packing defects could be dramatic. These defects should facilitate AH insertion, thereby increasing the scaffold-membrane friction, which would decrease scission time (see Equation 6). Perhaps more significantly, an increase in the degree of lipid chain unsaturation reduces the membrane edge tension γ (Evans et al., 2003), which will lower the energy barrier for nucleating a membrane pore before scission. Because the barrier decreases exponentially with decreasing edge tension (see Equation 6 and the associated discussion), lipid unsaturation could lower the scission time by orders of magnitude, making FDS relevant to fast scission in neurons.

### Conclusions

We have shown that in the presence of a pulling force, provided by motors or actin, BAR domain scaffolds enable scission through a generic friction-based mechanism. Whereas α helices contribute to destabilizing (severing) spherical buds, they are not required for FDS of tubes. The timescales associated with FDS are highly variable and can be adjusted by varying the membrane lipid composition, tuning FDS for different cellular contexts. Because BAR domains are ubiquitous, FDS is a basic and versatile scission mechanism that could occur throughout the cell and across cell types. This mechanism could, in principle, be extended to any protein that binds to membrane tubes, in which the resulting assembly imposes sufficient friction. Finally, this work could open new avenues of study into the competing tendencies of N-BAR proteins to stabilize or effect scission of transport intermediates.

## STAR★Methods

### Key Resources Table

**Table:** 

REAGENT or RESOURCE	SOURCE	IDENTIFIER
**Chemicals, Peptides, and Recombinant Proteins**		

Total brain extract lipids	Avanti Polar Lipids	cat# 131101P
L-α-phosphatidylinositol-4,5-bisphosphate (PI(4,5)P_2_,)	Avanti Polar Lipids	cat# 840046P
1,2-Dioleoyl-sn-glycero-3-phosphatidylcholine (DOPC)	Avanti Polar Lipids	cat# 850375
1,2-dioleoyl-sn-glycero-3-phosphatidylserine (DOPS)	Avanti Polar Lipids	cat# 840035
1,2-distearoyl-sn-glycero-3-phosphoethanolamine-N-[biotinyl(polyethylene glycol)-2000] (DSPE-PEG(2000)-biotin)	Avanti Polar Lipids	cat# 880129
1,2-dioleoyl-sn-glycero-3-phospho-L-serine-N-(7-nitro-2-1,3-benzoxadiazol-4-yl) (NBD-PS)	Avanti Polar Lipids	cat# 810198C
BODIPY-TR-C5-ceramide	Molecular Probes	cat# D7540
endophilin A2 N-BAR domain	McMahon lab (see STAR Methods)	N/A
β2 centaurin (BAR + PH domain)	McMahon lab (see STAR Methods)	N/A
endophilin A2 ΔH0	Johannes lab (see STAR Methods)	N/A
endophilin A2 E37K, D41K	McMahon lab (see STAR Methods)	N/A
full length endophilin A2 (N-BAR + SH3 domain)	Anne Schmidt lab	N/A
pGEX4T2	GE Healthcare	N/A
pGEX6P2	GE Healthcare	N/A
isopropyl β-D-1-thiogalactopyranoside	Sigma	cat# I5502
glutathione Sepharose	GE Healthcare	cat# 17075601
Thrombin	Serva	cat# 36402
S75 Sephadex	GE Healthcare	N/A
Alexa Fluor 488 C5 Maleimide	Life Technologies	cat# A10254
Strep-Tactin column	IBA	N/A
QHP column	GE Healthcare	N/A
streptavidin-coated polystyrene beads 3 μm	Spherotech	cat# SVP-30-5
β-casein from bovine milk (> 98%)	Sigma	cat# C6905
Tubulin	Manneville lab	N/A
biotinylated kinesin	Manneville lab	N/A
Taxol (paclitaxel)	Sigma	N/A
Imidazole	Sigma	I5513
ATP	Sigma	A1852
Dyngo-4a	Abcam	cat# AB120689
Ciliobrevin-D	Calbiochem	cat# 250401
Shiga toxin B-Subunit coupled to Alexa-A88 dye	Johannes lab	N/A
Hoechst 34580	Sigma Aldrich	cat# 63493
Alexa488-CTxB	Invitrogen	cat# C34775
2-deoxyglucose	Sigma Aldrich	cat# D8375
Sodium Azide	Sigma Aldrich	cat# S2002
HEPES	Mediatech	cat# 25-060-CI
BSA	Sigma Aldrich	A8806
Dulbecco’s modified Eagle medium (DMEM)	Life Technologies	cat# 61965-026
Fetal bovine serum (FBS)	PAN-Biotech	cat# 8500-P131704
Pen-Strep	Life Technologies	cat# 15140-122
Paraformaldehyde	Electron microscopy sciences	cat# 15710

**Experimental Models: Cell Lines**		

COS-7	ATCC	ATCC CRL-1651
HeLa C2TA	Johannes lab	N/A

**Software and Algorithms**		

MATLAB	The Mathworks	https://www.mathworks.com/products/matlab.html
Fiji	Fiji	https://fiji.sc/
Mathematica	Wolfram	https://www.wolfram.com/mathematica/

### Contact for Reagent and Resource Sharing

Further information and requests for resources and reagents should be directed to and will be fulfilled by the Lead Contact, Patricia Bassereau (pbassereau@curie.fr).

### Experimental Models and Subject Details

#### Cell lines

HeLa C2TA cells were cultured in Dulbecco’s modified Eagle medium (DMEM) complete supplemented with 10% Fetal Bovine Serum (FBS) + Pen-Strep antibiotics mixture (1x). The cells were cultured at 37°C in 5% CO_2_.

COS-7 cells were grown in containing 10% FBS at 37°C and 5% CO_2_. Cells were plated in Matek glass bottom culture plates two days prior to the experiments.

#### In vitro reconstituted membranes

Lipids used to reconstitute the cell membrane in vitro were purchased (see Key Resources Table) and stored at −80°C or aliquoted in CHCl_3_ and kept at −20°C. Once vesicles were prepared (see below), they were kept on ice and used within 3 hr.

### Method Details

#### Protein purification

Proteins used in this study were expressed and purified as part of our recent publication (Simunovic et al., 2016). We repeat the protocol here for completeness. Rat endophilin A2 WT (amino acids 1–247) and its mutant (E37K, D41K) were cloned into pGEX4T2. Mutations were made to create a clone with a single cysteine residue available for fluorescence labeling, to ensure the label does not interfere with membrane binding (C96A, C147A, and Q228C). Human β2 centaurin (amino acids 1–384) was cloned in pGEX6P2, mutating all cysteines (C10A, C42A, C53A, C156A, C321A, C316A, C329A, and C339A) and adding a new cysteine at the N terminus. GST-tagged proteins were expressed in BL21 DE3 bacteria at 18°C overnight after induction by 150 mM isopropyl β-D-1-thiogalactopyranoside, lysed in buffer (50 mM HEPES at pH = 7.4, 500 mM NaCl, 2 mM DTT, and protease inhibitor mixture) under high pressure (high pressure homogenizer; Constant Systems). The cleared lysate was incubated with glutathione Sepharose for 30 min at 4°C. The GST tag was cleaved off using thrombin or PreScission protease and the cleaved protein was passed over a Q Sepharose anion exchange column followed by a gel filtration column (S75 Sephadex). Endophilin A2 N-BAR domain and β2 centaurin were labeled with Alexa488 following the manufacturer’s protocol (A10254), concentrated, snap-frozen in liquid nitrogen, and stored at −80°C.

C-terminally strep-tagged mouse endoA2 ΔH0 was expressed in bacteria and purified on Strep-Tactin column as previously described (Renard et al., 2015). Eluates were loaded on a QHP column and eluted with a linear NaCl gradient in buffer (100 mM Tris-HCl at pH = 8.0, 1 mM EDTA). Fractions containing endoA2-Strep were then pooled and loaded on a Superdex 200 column for size exclusion chromatography. Protein purity was validated by SDS-polyacrylamide gel electrophoresis then endoA2 ΔH0-containing fractions were snap frozen in liquid nitrogen and stored at −80°C.

The full-length mouse endophilin A2 (N-BAR + SH3 domains) was a generous gift of Anne Schmidt, Institut Jacques Monod, Université Paris Diderot.

#### Preparation of giant unilamellar vesicles (GUVs)

GUVs were prepared by electroformation on Pt-wires under quasi-physiological salt conditions (Montes et al., 2007). First, we mixed CHCl_3_-solutions of the total brain extract and PI(4,5)P_2_ at 95:5 (molar ratio, molar mass of brain extract estimated to be 800 g.mol^−1^) to which we added 1% BODIPY-TR-C5-ceramide and ∼0.1% DSPE-PEG(2000)-biotin (both molar percent). The lipid mix was applied to a pair of Pt-wires, in drops separated by 0.5 cm (total ∼4 μL). The wires were dried under vacuum for 30–60 min then hydrated in a solution of 70 mM NaCl, 100 mM sucrose, and 10 mM tris, at pH = 7.4. We then applied AC current through the Pt-wires (assembled into a homemade Teflon chamber), using a functional generator, at 500 Hz and 280 mV overnight in the fridge. We disconnected the wires just prior to each experiment and used vesicles for no more than 4 hr. We collected vesicles directly from the wires using a pipette (∼10 μL of final solution per droplet of the lipid mix).

#### Preparation of supported bilayers

To make a supported lipid bilayer used in Figure S1, first the lipid mix composed of DOPC:DOPS (7:3, molar ratio) with 0.5% NBD-PS (molar percent), was dried under nitrogen to obtain 1 mg of dry mass. The mix was hydrated in 1 mL sucrose then extruded through a 100 nm polycarbonate filter. Thus-formed small vesicles were deposited onto an acid-cleaned coverslip to create a supported bilayer. The bilayer was rinsed with a solution of 100 mM NaCl and 10 mM tris buffer (pH = 7.4) and observed before and after adding the protein to a total bulk concentration of 3 μM using Nikon eclipse Ti inverted microscope.

#### Preparation of tensionless multilamellar lipid sheets

Multilamellar lipid sheets were prepared first by depositing a drop of the above-prepared brain extract–PI(4,5)P_2_ lipid mix onto an acid-cleaned glass slide. The deposit was dried for an hour under vacuum then rapidly hydrated with a solution of 100 mM NaCl and 10 mM tris buffer (pH = 7.4). This process creates multilamellar sheets with many tensionless tubules emanating from the edges. We imaged the edges of the sheet with differential interference contrast microscopy before and after adding the protein (to a bulk concentration of 3 μM) for up to 30 min.

#### Pulling tubes from GUVs

The experiment was carried out very similarly as described previously (Sorre et al., 2012). First, the tip of the aspiration pipette (∼5 μm in diameter at the tip) and the experimental chamber were immersed in a 5 g.L^−1^ solution of β-casein (dissolved in 100 mM NaCl, 10 mM tris, pH = 7.4) for 30 min to minimize the adhesion of lipids to the glass surface. The chamber was then rinsed several times and filled with the experimental solution (100 mM NaCl and 40 mM glucose, 10 mM tris, pH = 7.4). The ionic strength of solutions used to grow GUVs and for tubule-extrusion experiments was confirmed to be within 10 mOsm using an osmometer (Loser, Germany) to avoid osmotic shock. While theoretically the solutions inside and outside the vesicle can be varied (keeping their ionic strengths equal), the composition we used seemed optimal for tube-pulling experiments. We caution the reader not to exceed 40 mM in glucose concentration as we found it inhibits streptavidin-biotin interactions.

GUVs were directly collected from Pt-wires just prior to the experiment and a few μL of the GUV solution was added to the experimental chamber. A few μL of streptavidin-coated polystyrene beads 3 μm in diameter were added to the experimental chamber as well to a final bead concentration around 0.1x10−3% (w/v) or less.

Another pipette was filled with a solution of the protein (diluted in the experimental buffer to 1–5 μM monomeric concentration for all proteins except endoA2 ΔH0 where we used 7 μM monomeric concentration). The vesicles were left to deflate for 10–20 min after which we sealed the chamber with oil to prevent evaporation.

Vesicles with enough excess area to form an aspiration tongue were aspired in a micropipette, whose aspiration pressure sets membrane tension (Cuvelier et al., 2005, Kwok and Evans, 1981). By using a piezoactuator, we tethered the vesicle to a streptavidin-coated polystyrene bead trapped with optical tweezers, and gently pulled back, forming a tube between the vesicle and the bead. We observed the vesicle and the tube using confocal microscopy.

#### Fluorescence recovery after photobleaching (FRAP)

In the experiment, the lipid fluorophores in the ∼10-μm-long tube were bleached by imaging only the tube region at full laser power (∼ten images at a rate of 3 Hz; note that more than ten images runs the risk of photooxidation). Immediately after bleaching, the laser power was decreased and the system (vesicle + tube) imaged.

#### FDS by tube extension

We injected the protein near the tube at low pressure to avoid disturbing the system as descried previously (Sorre et al., 2012). After confirming the formation of a scaffold (force decrease and tube constriction, see Main text and (Simunovic et al., 2016)), we removed the injection pipette then applied a pulling force on the aspiration pipette, moving it away from the bead at a constant rate (50–8000 nm.s^−1^). We determined the average pulling rate from time-lapse confocal images.

#### FDS by kinesin motors in vitro

We followed a previously described protocol for the extrusion of tubules from GUVs (Leduc et al., 2010, Leduc et al., 2004). *Polymerization of microtubules.* 50 μL of tubulin (at ∼10 μM) was polymerized by incubation at 37°C for 15 min. We then added 2 μL of 1 mM taxol (diluted in water), which stabilizes the microtubules. We centrifuged the mix for 15 min at 37°C, at 70 000 rpm (ultracentrifuge, rotor TLA-100). We removed the supernatant and re-suspended the sediment in 50 μL BRB (25 μL of 4X BRB, 75 μL H_2_O, 3 μL of 1 mM taxol). We let the microtubules incubate for at least one day and we used them no more than 3 days after preparation. *Coupling kinesin to microtubules.* We assembled an experimental chambers using a glass slide and a coverslip, attached by melting a strip of parafilm, with a total volume between the slides of ∼5 μL. We filled the chamber with 5 μL polymerized microtubules and kept for 15 min at room temperature. Then, we incubated 5–10 μL of biotinylated kinesin (∼10 μM) with 5 μL streptavidin (at comparable concentration as kinesin) for 15 min on ice. During incubation, we first rinsed the experimental chamber (containing microtubules) with ∼10 μL of buffer composed of 97 μL of 50 mM imidazole (dissolved in 7 g.L^−1^ casein) and 3 μL of 1 mM taxol, buffered to pH ∼7. Importantly, the rinsing was done as carefully as possible, as fluxes in the chamber cause the polymerized microtubules to desorb from the glass. We incubated for 5 min at room temperature. Next, we rinsed (very carefully) with ∼10 μL of buffer composed of 96.5 μL of 50 mM imidazole, 0.5 μL of 1 M DTT, and 3 μL of 1 mM taxol, buffered to pH ∼7. We incubated for another 5 min. Finally, we injected the chamber with 5 μL kinesin that has been incubating with streptavidin. We incubated for at least 10 min. Note, this incubation step can be prolonged for a couple of hours if desired. *Kinesin-driven tube pulling.* To initiate the motors, we rinse the chamber with 10–15 μL motility buffer, composed of 89 μL experimental buffer (in our case, 40 mM glucose, 100 mM NaCl, and 10 mM tris, at pH = 7.4), 0.5 μL of 1 M DTT, 3 μL of 1 mM taxol, 2 μL of 100 mM ATP (freshly prepared), 3 μL of 4X oxygen scavenger (freshly prepared). In case the experimental buffer does not contain glucose, it needs to be added to the mix (2.5 μL of 1 M solution). Immediately after rinsing, 1–2 μL of highly concentrated solution of GUVs (prepared as described above) is added to the experimental chamber. After introducing GUVs, we tilt the chamber at 45° for one minute to help sediment the vesicles then mount it on the objective. We imaged as soon as possible. Note, that excessive exposure to fluorescent excitation can abort the motors due to oxidation.

#### FDS by dynein motors in vivo

To study the involvement of motor proteins that supply force needed to drive scission of STxB tubules in HeLa cells, we inhibited the activity of dynein motors using Ciliobrevin-D (100 μM, 30 min, 37°C). Dynein motors can interact with plasma membrane tubules induced by STxB and drive the tubule extension by pulling the membrane along existing microtubules (Day et al., 2015). To test the involvement of dynein in STxB tubule elongation dynamics in HeLa cells, we first inhibited the dynamin activity using Dyngo-4a (25 μM, 30 min, 37°C). In the presence of Dyngo-4a, STxB was localized in visible, micron-long tubular structures originating from plasma membrane (Figure S6A, panels ii, iv). Further, upon inhibition of dynein in dynamin-inhibited cells, the tubule lengths decreased almost back to those under control condition (Figure S6A, panels iii, iv), indicating the strong involvement of the dynein motor in pulling the STxB tubules to provide force for scission.

#### Theoretical model of FDS

We present here Supplemental Information a theoretical description and a model of FDS.

### Quantification and Statistical Analysis

#### Measuring membrane tension and force

The aspiration pressure in the pipette sets the membrane tension. At each tension step, the aspiration pressure is calculated from the hydrostatic pressure according to ΔP=ρgh, where ρ is the water density, g gravitational acceleration, and *h* the height of the water tank. Vesicle membrane tension, $\sigma_{\text{v}}$, is calculated using the Laplace equation: $\sigma_{\text{v}}=\Delta P\left(r_{\text{pip}}/2\left(1-r_{\text{pip}}/r_{\text{GUV}}\right)\right)$ where $r_{\text{pip}}$ and $r_{\text{GUV}}$ are the radius of the pipette and the GUV, respectively. At the same time, equilibrium membrane force (tube-retraction force) is calculated from the Hooke law: $f=k\left(a-a_{0}\right)$ where *a* is the average position of the bead during that measurement and *a*_0_ is the average position of the bead in the optical trap before pulling a tube. Both *a* and *a*_0_ are measured using videomicroscopy in bright field, while *k* is the stiffness of the optical trap (Sorre et al., 2012).

#### Measuring fluorescence intensity and tube radius

Fluorescence intensity of the membrane was taken by first fitting a line or a semicircle to, respectively, the maximum intensity segment of the tube or the GUV in confocal images (taken at the GUV equator), then calculating the mean intensity value along the fitted segment. Tube radius, *r*, can be measured from force and tension measurements (in the case of protein-free tubes) and from fluorescence (either protein-free or bound tubes). In Figure 2B, *r* is measured as $r=\sqrt{\kappa /2\sigma_{\text{v}}}$ (Derényi et al., 2002). In Figure S4, *r* at each step is measured as $r=10\;\text{nm}\;\times \;I_{\text{tub},\text{curr}}/I_{\text{tub},0}$, where $I_{\text{tub},\text{curr}}/I_{\text{tub},0}$ is the lipid fluorescence intensity ratio in the tube between the current and the initial time steps, while the prefactor 10 nm is the initial endoA2 scaffold radius, determined previously (Simunovic et al., 2016).

#### Measuring scaffold friction coefficient

The friction coefficient between the protein scaffold and membrane tube was determined in four separate ways. First, the pulling force versus time data for single experiments on endoA2 WT, endoA2 mut, and endoA2 ΔH0 were fitted with Equation 3 using the NonlinearModelFit function in Mathematica 11.0 (Figures 3B, 3G, and 3H). The error on the extracted fitting parameters represents the standard error; the scale of variance was determined using the dispersion of the data (i.e., from a weighted sum of squares). The results of these fits for multiple experiments on endoA2 WT (n = 10), endoA2 mut (n = 9), and endoA2 ΔH0 (n = 5) were compiled into a bar chart, in which the average values of the friction coefficient, ξ, are shown (Figure 3D); the error bars represent the SEM.

The second method to find ξ was to fit the force versus time data for endoA2 mut-covered tubes subject to a sudden change in tube length (Figures 3E and 3F). These data were fitted using equation S25, as described in the previous paragraph.

Next, the friction coefficient for endoA2 WT was also found from the scission times, $t_{\text{break}}$, as a function of pulling speed. These data, represented on log-lin scale, were fitted with Equation 6 using the LinearModelFit function in Mathematica, as described above; see Figure 4C. Finally, ξ was also obtained from the tube force at scission (minus the force before extension began), Δf, as a function of pulling speed. These datasets, for endoA2 WT, endoA2 mut, and endoA2 ΔH0, were fitted using Equation 5 (Figure 4F); a log-log representation of these data also allowed a fit of the exponent in the relation $\text{Δ}f\propto V^{\alpha }$, for which our model predicts α=1/3.

#### Quantifying tube breakage in FDS

Tube breakage in vitro was readily visible from confocal time-lapse images (e.g, Figure 1C). Alternatively, a sudden drop in membrane force to zero marks FDS. In observations of tube stabilization (under static conditions) or tube scission (upon tube extension), *n* values, as indicated in Main text, represent the total number of pulled tubes, where no more than one tube was pulled from a GUV.

CTxB tubulation assays were performed under ATP depleting conditions as previously described (Day et al., 2015). The cells were initially incubated in glucose-free DMEM containing 50 mM 2-deoxy-d-glucose, 0.02% sodium azide, 25 mM HEPES, and 1 mg/mL BSA for 15 min at 37°C and 5% CO_2_. They were rinsed twice and incubated for 5 min at room temperature with 100 nM Alexa 555 labeled CTxB. Subsequently, the cells were washed twice and imaged at 37°C in the ATP depleted media. Under these conditions, CTxB tubules typically remain attached to the plasma membrane but on average increase in length over time. We also observed that some tubules undergo complex motions including bidirectional motility.

Kymographs were generated for individual tubules using the Multiple Kymograph plugin of ImageJ (Fiji). The movement of the leading edge of each tubule was used as the marker of the position of the tubule. This was manually tracked as a function of time in each of the kymographs. The tubule trajectories were then divided into segments based on directionality for velocity analysis. A change in direction was defined as a point at which the slope of the trajectories change and each slope represents a single instantaneous velocity of the tubule (25 tubules from 6 separate movies were chosen for analysis of tubule velocities and 228 instantaneous velocities were measured).

Quantification of STxB-induced tubule length under various experimental conditions (shown in Figure S6A) was done as previously described in (Renard et al., 2015). Tube recognition was done using a Fiji macro, which enhanced the tubular structures by computing eigenvalues of the Hessian matrix on Gaussian-filtered images (with sigma = 1 pixel), as implemented in the tubeness plugin. The threshold for tubules was done such that structures containing less than three pixels were discarded. If necessary, a manual correction of segmented tubules was performed upon a visual check. The tube-segmented structures were then reduced to a one-pixel-thick skeleton, using the Fiji plugin skeletonize. The pixel length of skeletonized tubules was then converted to actual length. In Figure S6A: ^∗∗∗^p < 0.001 (One-way Anova test). Data are mean ± SEM of two independent experiments (n = 25 cells per condition).

## Author Contributions

M.S., J.-B.M., K.R., and D.B. performed and analyzed experiments. M.S. and P.B. conceived experiments. A.C.-J. and J.P. conceived and developed the theory. P.B. secured funding. H.-F.R. and E.E. provided proteins. H.-F.R., E.E., K.R., A.K.K., G.A.V., H.T.M., and L.J. provided conceptual advice and feedback. M.S., P.B., and A.C.-J. wrote the manuscript. All authors discussed the results and commented on the manuscript.

## Supplemental Information

Document S1. Theoretical model of FDS and Tables S1 and S2

Movie S1. EndoA2 N-BAR Domain Does Not Induce Scission of Tubular Membranes, Related to Figure 1 and Figure S1 Confocal fluorescence time-lapse during injection of an N-BAR domain of endoA2 near a tube pulled from a micropipette-aspired GUV. Movie shows spontaneous tubulation of the GUV and the formation of a scaffold on the tube (causing it to buckle). No scission is observed under these conditions. Red, lipids; green, NBAR domain.

Movie S2. FDS of a Tube Partially Scaffolded by EndoA2, Related to Figure 1 Movie shows extension of a tube partially scaffolded by endoA2 N-BAR domain leading up to scission at the tube-GUV interface. Shown are the confocal fluorescence time lapse (left) and the tube retraction force, *f*, both changing with time, *t*. Top left: N-BAR (green); center left: lipids (red); bottom left: overlay

Movie S3. FDS of a Tube Fully Scaffolded by EndoA2, Related to Figure 1 Movie shows extension of a tube initially fully scaffolded by endoA2 N-BAR domain leading up to scission. After initial extension, gaps form in the scaffold making fully and partially scaffolded tubes equivalent in FDS. Shown are confocal fluorescence time lapse (left) and tube retraction force, *f*, both changing with time, *t*. Top left: N-BAR (green); center left: lipids (red); bottom left: overlay

Movie S4. FDS by Kinesin, Related to Figure 5 and Figure S6 Confocal fluorescence time lapse showing two scission events of kinesin-pulled tubes connected to a GUV seconds after endoA2 injection into the system. Scale bar, 2 μm.

Movie S5. FDS by Kinesin, Related to Figure 5 and Figure S6 Confocal fluorescence time lapse showing a scission event of a kinesin-pulled tube connected to a GUV seconds after endoA2 injection into the system. Shown is a different example from Movie S4. Scale bar, 2 μm.

## References

[bib1] Allain J.M., Storm C., Roux A., Ben Amar M., Joanny J.F. (2004). Fission of a multiphase membrane tube. Phys. Rev. Lett..

[bib2] Ambroso M.R., Hegde B.G., Langen R. (2014). Endophilin A1 induces different membrane shapes using a conformational switch that is regulated by phosphorylation. Proc. Natl. Acad. Sci. USA.

[bib3] Berk D.A., Clark A., Hochmuth R.M. (1992). Analysis of lateral diffusion from a spherical cell surface to a tubular projection. Biophys. J..

[bib4] Boucrot E., Pick A., Çamdere G., Liska N., Evergren E., McMahon H.T., Kozlov M.M. (2012). Membrane fission is promoted by insertion of amphipathic helices and is restricted by crescent BAR domains. Cell.

[bib5] Boucrot E., Ferreira A.P., Almeida-Souza L., Debard S., Vallis Y., Howard G., Bertot L., Sauvonnet N., McMahon H.T. (2015). Endophilin marks and controls a clathrin-independent endocytic pathway. Nature.

[bib6] Boulant S., Kural C., Zeeh J.C., Ubelmann F., Kirchhausen T. (2011). Actin dynamics counteract membrane tension during clathrin-mediated endocytosis. Nat. Cell Biol..

[bib7] Brochard-Wyart F., Borghi N., Cuvelier D., Nassoy P. (2006). Hydrodynamic narrowing of tubes extruded from cells. Proc. Natl. Acad. Sci. USA.

[bib8] Callan-Jones A., Durand M., Fournier J.-B. (2016). Hydrodynamics of bilayer membranes with diffusing transmembrane proteins. Soft Matter.

[bib9] Campelo F., McMahon H.T., Kozlov M.M. (2008). The hydrophobic insertion mechanism of membrane curvature generation by proteins. Biophys. J..

[bib10] Campillo C., Sens P., Köster D., Pontani L.L., Lévy D., Bassereau P., Nassoy P., Sykes C. (2013). Unexpected membrane dynamics unveiled by membrane nanotube extrusion. Biophys. J..

[bib11] Chen Z., Zhu C., Kuo C.J., Robustelli J., Baumgart T. (2016). The N-terminal amphipathic helix of endophilin does not contribute to its molecular curvature generation capacity. J. Am. Chem. Soc..

[bib12] Cuvelier D., Derényi I., Bassereau P., Nassoy P. (2005). Coalescence of membrane tethers: experiments, theory, and applications. Biophys. J..

[bib13] Day C.A., Baetz N.W., Copeland C.A., Kraft L.J., Han B., Tiwari A., Drake K.R., De Luca H., Chinnapen D.J., Davidson M.W. (2015). Microtubule motors power plasma membrane tubulation in clathrin-independent endocytosis. Traffic.

[bib14] Derényi I., Jülicher F., Prost J. (2002). Formation and interaction of membrane tubes. Phys. Rev. Lett..

[bib15] Domanov Y.A., Aimon S., Toombes G.E., Renner M., Quemeneur F., Triller A., Turner M.S., Bassereau P. (2011). Mobility in geometrically confined membranes. Proc. Natl. Acad. Sci. USA.

[bib16] Dommersnes P., Orwar O., Brochard-Wyart F., Joanny J. (2005). Marangoni transport in lipid nanotubes. Europhys. Lett..

[bib17] Evans E., Yeung A. (1994). Hidden dynamics in rapid changes of bilayer shape. Chem. Phys. Lipids.

[bib18] Evans E., Heinrich V., Ludwig F., Rawicz W. (2003). Dynamic tension spectroscopy and strength of biomembranes. Biophys. J..

[bib19] Ferguson S.M., Raimondi A., Paradise S., Shen H., Mesaki K., Ferguson A., Destaing O., Ko G., Takasaki J., Cremona O. (2009). Coordinated actions of actin and BAR proteins upstream of dynamin at endocytic clathrin-coated pits. Dev. Cell.

[bib20] Grassart A., Cheng A.T., Hong S.H., Zhang F., Zenzer N., Feng Y., Briner D.M., Davis G.D., Malkov D., Drubin D.G. (2014). Actin and dynamin2 dynamics and interplay during clathrin-mediated endocytosis. J. Cell Biol..

[bib21] Johannes L., Parton R.G., Bassereau P., Mayor S. (2015). Building endocytic pits without clathrin. Nat. Rev. Mol. Cell Biol..

[bib22] Kirchhausen T., Owen D., Harrison S.C. (2014). Molecular structure, function, and dynamics of clathrin-mediated membrane traffic. Cold Spring Harb. Perspect. Biol..

[bib23] Koster G., VanDuijn M., Hofs B., Dogterom M. (2003). Membrane tube formation from giant vesicles by dynamic association of motor proteins. Proc. Natl. Acad. Sci. USA.

[bib24] Kozlovsky Y., Kozlov M.M. (2003). Membrane fission: model for intermediate structures. Biophys. J..

[bib25] Kramers H.A. (1940). Brownian motion in a field of force and the diffusion model of chemical reactions. Physica.

[bib26] Kwok R., Evans E. (1981). Thermoelasticity of large lecithin bilayer vesicles. Biophys. J..

[bib27] Leduc C., Campàs O., Zeldovich K.B., Roux A., Jolimaitre P., Bourel-Bonnet L., Goud B., Joanny J.F., Bassereau P., Prost J. (2004). Cooperative extraction of membrane nanotubes by molecular motors. Proc. Natl. Acad. Sci. USA.

[bib28] Leduc C., Campàs O., Joanny J.F., Prost J., Bassereau P. (2010). Mechanism of membrane nanotube formation by molecular motors. Biochim. Biophys. Acta.

[bib29] Liu J., Kaksonen M., Drubin D.G., Oster G. (2006). Endocytic vesicle scission by lipid phase boundary forces. Proc. Natl. Acad. Sci. USA.

[bib30] Llobet A., Gallop J.L., Burden J.J., Camdere G., Chandra P., Vallis Y., Hopkins C.R., Lagnado L., McMahon H.T. (2011). Endophilin drives the fast mode of vesicle retrieval in a ribbon synapse. J. Neurosci..

[bib31] Massol R.H., Boll W., Griffin A.M., Kirchhausen T. (2006). A burst of auxilin recruitment determines the onset of clathrin-coated vesicle uncoating. Proc. Natl. Acad. Sci. USA.

[bib32] McMahon H.T., Boucrot E. (2011). Molecular mechanism and physiological functions of clathrin-mediated endocytosis. Nat. Rev. Mol. Cell Biol..

[bib33] Meinecke M., Boucrot E., Camdere G., Hon W.C., Mittal R., McMahon H.T. (2013). Cooperative recruitment of dynamin and BIN/amphiphysin/Rvs (BAR) domain-containing proteins leads to GTP-dependent membrane scission. J. Biol. Chem..

[bib34] Merkel R., Sackmann E., Evans E. (1989). Molecular friction and epitactic coupling between monolayers in supported bilayers. J. Phys..

[bib35] Merrifield C.J., Kaksonen M. (2014). Endocytic accessory factors and regulation of clathrin-mediated endocytosis. Cold Spring Harb. Perspect. Biol..

[bib36] Montes L.R., Alonso A., Goñi F.M., Bagatolli L.A. (2007). Giant unilamellar vesicles electroformed from native membranes and organic lipid mixtures under physiological conditions. Biophys. J..

[bib37] Mooren O.L., Galletta B.J., Cooper J.A. (2012). Roles for actin assembly in endocytosis. Annu. Rev. Biochem..

[bib38] Morlot S., Galli V., Klein M., Chiaruttini N., Manzi J., Humbert F., Dinis L., Lenz M., Cappello G., Roux A. (2012). Membrane shape at the edge of the dynamin helix sets location and duration of the fission reaction. Cell.

[bib39] Neumann S., Schmid S.L. (2013). Dual role of BAR domain-containing proteins in regulating vesicle release catalyzed by the GTPase, dynamin-2. J. Biol. Chem..

[bib40] Peter B.J., Kent H.M., Mills I.G., Vallis Y., Butler P.J., Evans P.R., McMahon H.T. (2004). BAR domains as sensors of membrane curvature: the amphiphysin BAR structure. Science.

[bib41] Pinot M., Vanni S., Pagnotta S., Lacas-Gervais S., Payet L.A., Ferreira T., Gautier R., Goud B., Antonny B., Barelli H. (2014). Lipid cell biology. Polyunsaturated phospholipids facilitate membrane deformation and fission by endocytic proteins. Science.

[bib42] Rawicz W., Olbrich K.C., McIntosh T., Needham D., Evans E. (2000). Effect of chain length and unsaturation on elasticity of lipid bilayers. Biophys. J..

[bib43] Renard H.F., Simunovic M., Lemière J., Boucrot E., Garcia-Castillo M.D., Arumugam S., Chambon V., Lamaze C., Wunder C., Kenworthy A.K. (2015). Endophilin-A2 functions in membrane scission in clathrin-independent endocytosis. Nature.

[bib44] Römer W., Pontani L.L., Sorre B., Rentero C., Berland L., Chambon V., Lamaze C., Bassereau P., Sykes C., Gaus K., Johannes L. (2010). Actin dynamics drive membrane reorganization and scission in clathrin-independent endocytosis. Cell.

[bib45] Roux A., Cappello G., Cartaud J., Prost J., Goud B., Bassereau P. (2002). A minimal system allowing tubulation with molecular motors pulling on giant liposomes. Proc. Natl. Acad. Sci. USA.

[bib46] Roux A., Uyhazi K., Frost A., De Camilli P. (2006). GTP-dependent twisting of dynamin implicates constriction and tension in membrane fission. Nature.

[bib47] Schmid S.L., Sorkin A., Zerial M. (2014). Endocytosis: past, present, and future. Cold Spring Harb. Perspect. Biol..

[bib48] Sciaky N., Presley J., Smith C., Zaal K.J., Cole N., Moreira J.E., Terasaki M., Siggia E., Lippincott-Schwartz J. (1997). Golgi tubule traffic and the effects of brefeldin A visualized in living cells. J. Cell Biol..

[bib49] Shnyrova A.V., Bashkirov P.V., Akimov S.A., Pucadyil T.J., Zimmerberg J., Schmid S.L., Frolov V.A. (2013). Geometric catalysis of membrane fission driven by flexible dynamin rings. Science.

[bib50] Simunovic M., Mim C., Marlovits T.C., Resch G., Unger V.M., Voth G.A. (2013). Protein-mediated transformation of lipid vesicles into tubular networks. Biophys. J..

[bib51] Simunovic M., Evergren E., Golushko I., Prévost C., Renard H.F., Johannes L., McMahon H.T., Lorman V., Voth G.A., Bassereau P. (2016). How curvature-generating proteins build scaffolds on membrane nanotubes. Proc. Natl. Acad. Sci. USA.

[bib52] Skjeldal F.M., Strunze S., Bergeland T., Walseng E., Gregers T.F., Bakke O. (2012). The fusion of early endosomes induces molecular-motor-driven tubule formation and fission. J. Cell Sci..

[bib53] Sorre B., Callan-Jones A., Manzi J., Goud B., Prost J., Bassereau P., Roux A. (2012). Nature of curvature coupling of amphiphysin with membranes depends on its bound density. Proc. Natl. Acad. Sci. USA.

[bib54] Soykan T., Maritzen T., Haucke V. (2016). Modes and mechanisms of synaptic vesicle recycling. Curr. Opin. Neurobiol..

[bib55] Sundborger A., Soderblom C., Vorontsova O., Evergren E., Hinshaw J.E., Shupliakov O. (2011). An endophilin-dynamin complex promotes budding of clathrin-coated vesicles during synaptic vesicle recycling. J. Cell Sci..

[bib56] Takei K., Slepnev V.I., Haucke V., De Camilli P. (1999). Functional partnership between amphiphysin and dynamin in clathrin-mediated endocytosis. Nat. Cell Biol..

[bib57] Taylor M.J., Lampe M., Merrifield C.J. (2012). A feedback loop between dynamin and actin recruitment during clathrin-mediated endocytosis. PLoS Biol..

[bib58] Traer C.J., Rutherford A.C., Palmer K.J., Wassmer T., Oakley J., Attar N., Carlton J.G., Kremerskothen J., Stephens D.J., Cullen P.J. (2007). SNX4 coordinates endosomal sorting of TfnR with dynein-mediated transport into the endocytic recycling compartment. Nat. Cell Biol..

[bib59] Yang H.J., Sugiura Y., Ikegami K., Konishi Y., Setou M. (2012). Axonal gradient of arachidonic acid-containing phosphatidylcholine and its dependence on actin dynamics. J. Biol. Chem..

[bib60] Yoshida Y., Kinuta M., Abe T., Liang S., Araki K., Cremona O., Di Paolo G., Moriyama Y., Yasuda T., De Camilli P., Takei K. (2004). The stimulatory action of amphiphysin on dynamin function is dependent on lipid bilayer curvature. EMBO J..

