# Supplementary material for: Friction Mediates Scission of Tubular Membranes Scaffolded by BAR Proteins
Source: Cell. 2017 Jun 29;170(1):172–184.e11. doi: 10.1016/j.cell.2017.05.047 (PMC5576516; doi:10.1016/j.cell.2017.05.047)
Supplement: Document S1. Theoretical model of FDS and Tables S1 and S2 [file mmc1.pdf]

Cell, Volume *170*

Supplemental Information

Friction Mediates Scission of Tubular Membranes Scaffolded by BAR Proteins

Mijo Simunovic, Jean-Baptiste Manneville, Henri-François Renard, Emma Evergren, Krishnan Raghunathan, Dhiraj Bhatia, Anne K. Kenworthy, Gregory A. Voth, Jacques Prost, Harvey T. McMahon, Ludger Johannes, Patricia Bassereau, and Andrew Callan-Jones

Supplemental Results

Theoretical model of FDS

1 Introduction

We present here a theoretical description and a model of FDS. Our experimental measurements indicate that the force holding a membrane tube coated by a protein scaffold increases with time upon elongation, in contrast with protein-free tubes, for which the force is constant as its length changes (Derenyi et al., 2002; Koster et al., 2005). Combining these observations with the reduction in lipid mobility detected by FRAP that we found, we propose that scaffold/membrane friction opposes the relative movement of these two constituents. Based on this hypothesis, we present first a model of the tube force (Sec. 2), and then use this as input into a model of tube scission based on membrane pore nucleation (Sec. 3).

2 Theory of the force on a protein scaffolded membrane tube

When protein-scaffolded tubes were extended at constant speed V, we found that the force, $f(t)$, increased at short times after extension began, and then tended to saturate. The saturating force, f_∞, was seen to increase with V. These observations suggest a viscoelastic-like response: at short times the behavior is elastic, as lipid flow from the vesicle to the tube is impeded by friction, the tube radius decreases, and f increases due to greater bending energy of the tubular membrane. At longer times, a balance between tube extension and lipid influx underneath the scaffold sets in, and the force becomes friction-dominated.

2.1 Force on an extended tube

To model these behaviors, we consider a tube of length $L_{\rm tube}(t)$ coated with a protein scaffold of fixed radius $r_{\rm s}$; see Figure S5. Since tubes were often found to be incompletely coated (see, for instance, Figure 1B), the total length is written $L_{\rm tube}(t) = L_{\rm s} + L(t)$, where $L_{\rm s}$ and $L(t)$ are the lengths of scaffolded and un-scaffolded tubes (Figure 3A). The un-scaffolded tube is expected to be cylindrical, with radius $r(t)$, at distances of the order r away from the scaffold interface (Morlot et al., 2012). In cases where tubes appeared to be fully covered before extension began, such as for the tubes pulled at 50 nm.s^{-1} and 0.98 μm.s^{-1} (Figure 3B), we found that almost immediately after, gaps in the scaffold appeared (Figure S4 and Movie S3). This effectively renders the tubes incompletely coated for most of the extension period, and our hypothesis of partially coated tubes is generally valid. Upon extension, we assume that the scaffold is rigid and does not change length (see Figure 1B), and therefore $L(t) = L_0 + \Delta L(t)$, with L_0 the initial length of uncoated tube and $\Delta L(t)$ the controlled change in tube length. For constant speed extension $\Delta L(t) = Vt$ and for a sudden step, $\Delta L(t) = \Delta L_{\rm step}$.

Changing the length of a scaffolded tubes results in a time-dependent force, $f(t)$, which can be obtained by combining the following basic elements:

- First, the length of the bare part of tube is $L(t) = L_0 + \Delta L(t)$, where

$$\Delta L(t) = \begin{cases} Vt, & \text{constant speed elongation} \\ \Delta L_{\rm step}, & \text{sudden step elongation}\,. \end{cases} \qquad \text{S1}$$

- As the tube is extended, the tension in the uncoated part of the tube is expected to increase. The tube tension is given by $\sigma(t) = K_A(A - A_0)/A_0$, where K_A is the area compressibility modulus of the membrane, $A(t)$ is the uncoated tube area, and $A_0(t)$ is the relaxed, or “preferred", uncoated area (Evans et al., 1976). During extension, $A(t)$ changes according to $dA/dt = 2\pi d(rL)/dt$. The relaxed area $A_0(t)$ changes as a result of the flux of lipids from the vesicle with speed v_l underneath the scaffold, and thus $dA_0/dt = 2\pi r_{\rm s} v_l$. Accordingly, the time derivative of σ is

$$\frac{d\sigma}{dt} = \frac{K_A}{A}\left[\frac{d}{dt}(2\pi r L) - 2\pi\, r_{\rm s}\, v_l\right]\,. \qquad \text{S2}$$

- The total force acting along a cross-section of partially scaffolded tube is uniform, and therefore equal

to the force acting on a cross-section of bare tube. This force is related to $\sigma(t)$ and the radius $r(t)$ (Dommersnes et al., 2005):

$$f = \pi\sigma r + \frac{3\pi\kappa}{2r}\,, \tag{S3}$$

where κ is the membrane bending modulus. Note that, in assuming a uniform f, we have neglected the drag on the tube exerted by the surrounding solution. More precisely, the drag on a tube of length L and radius r is $f_{\text{drag}} \simeq 2\pi\eta L\,V/\ln{(L/r)}$, where η is the solution viscosity (Keller and Rubinow, 1976). Taking $\eta = 10^{-3}$ Pa.s (water), $L = 5$ μm, $r = 10$ nm, and $V = 6$ μm/s (the maximum pulling speed), we find $f_{\text{drag}} \approx 0.03$ pN, and thus negligible compared to the forces that we measure.

- The bare tube radius r is in turn given by a Laplace-like law across the tube membrane:

$$\Delta P = \frac{\sigma}{r} - \frac{\kappa}{2r^3}\,. \tag{S4}$$

Here, ΔP is the pressure difference between the tube interior and exterior: $\Delta P = P_{\text{t}} - P_{\text{e}}$. Since the connecting vesicle is very large, the pressure inside it is $P_{\text{v}} \approx P_{\text{e}}$, and therefore ΔP is also the pressure difference between inside the tube and inside the vesicle. It is this pressure difference that drives the flux of interior liquid during tube length changes.

- As the tube is extended, the rate of change in volume of the bare part of the tube must be equal to the liquid flux through the scaffolded region (assuming constant scaffold length):

$$\frac{d}{dt}\left(\pi r^2 L\right) = Q\,, \tag{S5}$$

where Q is the flux. Assuming Poiseuille flow underneath the scaffold, with liquid velocity v_l at $r = r_{\text{s}}$, Q is given by

$$Q = \pi\, r_{\text{s}}^2 \left[v_l - \frac{r_{\text{s}}^2}{8\eta}\frac{\Delta P}{L_{\text{s}}}\right]\,, \tag{S6}$$

- The final ingredient requires specifying a relation between the tube tension, the lipid speed v_l, and the scaffold/membrane friction. Assuming that the scaffold is mechanically coupled to the pipette (confirmed by Figure 1B), pulling on the tube generates friction between the bilayer and the coat.

We propose that the tension along the bare part of the tube is

$$\sigma = \sigma_0 + \xi\, v_l\,, \tag{S7}$$

where σ_0 is the tube tension before elongation begins and ξ is the scaffold/membrane friction coefficient. Note that Equation S7 can be understood from a semi-microscopic picture in which relative movement between membrane lipids and the scaffold results in a surface shear stress; see (Merkel et al., 1989) and further discussion in Sec. 5.

Equations S2–S7 can be solved numerically for f, σ, r, ΔP, Q and v_l. However, ΔP relaxes to zero very quickly after elongation begins, and as a result

$$r(t) \simeq \sqrt{\frac{\kappa}{2\sigma(t)}}\,, \tag{S8}$$

which is just the equilibrium bare tube radius at a tension σ (Derenyi et al., 2002). The quick relaxation of ΔP can be seen by combining Eqs. S5-S6:

$$\frac{8\eta L_\mathrm{s}}{r_\mathrm{s}^4}\frac{d}{dt}\left(r^2 L\right) = \frac{8\eta L_\mathrm{s}\, v_l}{r_\mathrm{s}^2} - \Delta P\,. \tag{S9}$$

The lefthand side and the first term on the right are both of order $8\eta L_\mathrm{s} V/r_\mathrm{s}^2 \sim 80$ Pa, assuming $\eta = 10^{-3}$ Pa·s, $L_\mathrm{s} = 1\ \mu$m, $V = 1\ \mu$m/s, and $r_\mathrm{s} = 10$ nm. However, the two terms contributing to ΔP [Eq. S4] are both of order $\sigma/r \sim 10^4 - 10^5$ Pa, assuming $\sigma = 10^{-4} - 10^{-3}$ N/m and $\kappa = 45\, k_\mathrm{B}T$ (k_B is Boltzmann's constant and T is temperature). Thus, the terms in ΔP must balance, which leads to the expression for $r(t)$, Eq. S8.

As a result of this simplication, the tube force, Eq. S3, can be written in two ways:

$$f(t) = \frac{2\pi\kappa}{r(t)} \tag{S10a}$$

$$f(t) = 2\pi\sqrt{2\kappa\sigma(t)}\,. \tag{S10b}$$

Combining these expressions with Eqs. S2 and S7 yields an autonomous equation for $f(t)$:

$$\frac{1}{8\pi^2\kappa}\frac{d}{dt}\left(f^2\right) = \frac{2\pi K_A}{A}\left[2\pi\kappa\frac{d}{dt}\left(\frac{L}{f}\right) - \frac{f^2 - f_0^2}{8\pi^2\kappa\,\xi/r_\mathrm{s}}\right]\,, \tag{S11}$$

where $f_0 = 2\pi\sqrt{2\kappa\sigma_0}$ is the tube force before elongation begins.

A final simplification can be made. Comparing the df/dt terms on the left and right sides above shows that the lefthand side is negligible for forces $f < \left(\frac{32\pi^4\kappa^2 K_A L}{A}\right)^{1/3}$. Taking $K_A = 200$ mN/m (Rawicz et al., 2000), $A = 2\pi r L \sim 0.3\ \mu\text{m}^2$, and $L = 5\ \mu$m, we find that this condition requires $f \lesssim 700$ pN, which is much larger than the forces that we measure. Thus, the terms on the righthand side above must balance, implying $A(t) \simeq A_0(t)$, or that the membrane tube is essentially incompressible. As a result, we obtain

$$\frac{d}{dt}\left(\frac{L}{f}\right) = \frac{r_\text{s}}{16\pi^3\kappa^2\xi}\left(f^2 - f_0^2\right) . \tag{S12}$$

Equation S12 is the central equation of our model and can be solved to find f for different pulling protocols, such as given by Eq. S1.

2.2 Constant speed elongation

For a constant speed elongation experiment [Eq. S1], we see directly from Eq. S12 that at long times after elongation begins, f saturates to a value f_∞ obtained by solving the cubic equation

$$f^3 - f_0^2\, f - 16\pi^3\kappa^2\xi\, V/r_\text{s} = 0\,, \tag{S13}$$

and thus, for large V,

$$f_\infty \simeq \left(16\pi^3\kappa^2\xi\, V/r_\text{s}\right)^{1/3}\ ; \tag{S14}$$

see also Eq. 5 in the main text. Equation S13 is almost identical to an equation obtained for pulling tubes from plasma membranes that experience friction with membrane-cortex linking proteins (Brochard-Wyart et al., 2006). Note that in the problem studied in (Brochard-Wyart et al., 2006), the tube is bare, its radius is uniform, and thus every point on it moves at the same speed, equal to the constant pulling speed V; as a result, the force is time-independent. In our case, however, the tube scaffold friction prevents lipids underneath it from flowing instantly when elongation begins, and thus initially $v_l = 0$. Gradually, v_l increases, giving rise to the time dependence of f. Finally, we note that the scaling of f_∞ with $V^{1/3}$ can be understood simply since $f^2 \propto \sigma \propto v_l$, while by mass conservation at steady state, $v_l = V r_\infty / r_\text{s}$, with a limiting value of the uncoated tube radius $r_\infty \propto 1/f_\infty$ [Eq. S10a]; therefore, $f_\infty^3 \propto V$, or $f_\infty \propto V^{1/3}$.

Equation S12 can be integrated for $L = L_0 + Vt$, and after considerable algebra an implicit expression

for f can be obtained. Defining the characteristic time

$$\tau_{\mathrm{r}} = \frac{16\pi^3\kappa^2\xi L_0}{r_{\mathrm{s}} f_0^3} \tag{S15}$$

(it will be seen later to be related to the force relaxation time after a step length jump) and re-scaling time, pulling speed, and force according to $t' = t/\tau_{\mathrm{r}}$, $V' = V\tau_{\mathrm{r}}/L_0$, and $f' = f/f_0$, Eq. S12 can be re-written as

$$\frac{\dot{f}'}{f'} = -\frac{f'^3 - f' - V'}{1 + V't'}\,, \tag{S16}$$

where the overdot denotes differentiation with respect to t'. After some calculation we find

$$f' \prod_{n=1}^{3} \left(\frac{1 - a_n}{f' - a_n}\right)^{\frac{a_n^2 - 1}{3a_n^2 - 1}} = 1 + V't'\,, \tag{S17}$$

where

$$\begin{aligned} a_1 &= \frac{2 \cdot 3^{1/3} + 2^{1/3}\alpha^{2/3}}{6^{2/3}\alpha^{1/3}} \\ a_2 &= -\frac{1 + i\sqrt{3}}{2^{1/3}3^{1/3}\alpha^{1/3}} - \frac{\left(1 - i\sqrt{3}\right)\alpha^{1/3}}{2^{4/3} \cdot 3^{2/3}} \\ a_3 &= a_2^*\,. \end{aligned} \tag{S18}$$

In the above, $i = \sqrt{-1}$, the asterisk denotes complex conjugation, and

$$\alpha = 9V' + \sqrt{81V'^2 - 12}\,. \tag{S19}$$

Fortunately a useful simplification can be made, since $\alpha \gg 1$—that is, even at the lowest pulling speed, roughly 50 nm/s, $V' = 16\pi^3\kappa^2\xi\, V/(r_{\mathrm{s}} f_0^3) \approx 3$, assuming $f_0 = 10$ pN, $\xi = 50$ Pa.s (comparable to measured values; see Table S2), and thus $\alpha \approx 50$. In the limit $\alpha \gg 1$, Eq. S17 can be shown to simplify to the cubic equation

$$f'^3 - a(t')f' - V'a(t') = 0 \tag{S20}$$

where $a(t') = \left[1 + V'/(1 + V't')^3\right]^{-1}$. The positive real root to this equation can be readily found, though its expression in terms of t' and V' is cumbersome. Instead, a very good approximate formula

valid for $V' \gg 1$ can be obtained, namely $f' \simeq (V'a)^{1/3}$, which leads to

$$f(t) = f_0 \frac{1 + Vt/L_0}{\left[1 + \frac{(1+Vt/L_0)^3}{16\pi^3\kappa^2\xi V/(r_s f_0^3)}\right]^{1/3}} ; \qquad \text{S21}$$

see also Equation (3) of the main text. Equation S21 reveals two distinct force regimes that are consistent with our experiments: for times $t < t^*$, where

$$t^* = \left(\frac{16\pi^3\kappa^2\xi L_0^3}{r_s f_0^3 V^2}\right)^{1/3} , \qquad \text{S22}$$

f increases from f_0 linearly with time, whereas for $t > t^*$ tends to saturate to f_∞. Equation S21 was then used to fit experimental force data (Figure 3), allowing us to extract the friction coefficient ξ for endoA2 WT, endo ΔH0, and endo mut. Note that the bare tube length prior to elongation, L_0, is difficult to measure experimentally, and was handled as a second fit parameter.

2.3 Force relaxation after a jump in tube length

How the tube force relaxes after a sudden change in tube length, ΔL_{step}, provides a second way to probe scaffold/membrane friction. Prior to the length change, assumed to occur at $t = t_0$, we have $L = L_0$ and $f = f_0$; right after the jump, $L = L_0 + \Delta L_{\text{step}}$ and $f = f_{\text{peak}}$. The peak force, f_{peak} is found by integrating Eq. S12 from $t = t_0 - \epsilon$ to $t_0 + \epsilon$, with ϵ the short period over which the step is applied. This leads to

$$\frac{L_0}{f_0} = \frac{L_0 + \Delta L_{\text{step}}}{f_{\text{peak}}} . \qquad \text{S23}$$

Experimentally, ΔL_{step} is controlled and f_{peak} can be measured directly, whereas L_0 is unknown. The above equation, however, can be solved to infer L_0:

$$L_0 = \frac{\Delta L_{\text{step}}}{f_{\text{peak}}/f_0 - 1} . \qquad \text{S24}$$

With this knowledge, the force relaxation curve can be calculated by solving the following variant of Eq. S12:

$$\frac{df}{dt} = -\frac{r_s}{16\pi^3\kappa^2\xi(L_0 + \Delta L_{\text{step}})} f^2 \left(f^2 - f_0^2\right) , \qquad \text{S25}$$

with the initial condition $f(t_0) = f_{\text{peak}}$. While this differential equation is non-linear, for small $\Delta L_{\text{step}} = \delta L$, we can write $f = f_0 + \delta f$ and the righthand side above can be linearized, yielding

$$\frac{d\delta f}{dt} \simeq -\frac{2}{\tau_{\text{r}}}\delta f\,, \qquad \text{S26}$$

where the characteristic time τ_{r}, given by Eq. S15, appears. Therefore, this quantity is closely related to the relaxation time constant for the force after a sudden length change. Equation S25 was then used to fit our force relaxation data, yielding another determination of ξ.

3 Model of tube scission through membrane pore nucleation

3.1 Energy barrier for pore nucleation

In this last section, we develop a model of tube scission resulting from the friction-driven force increase discussed above. The proposed scission mechanism involves membrane pore nucleation and growth at sufficient tension, a process which has been studied in synthetic membrane systems (Evans et al., 2003). When extended at constant speed V, we found that endoA2-coated tubes broke at a time t_{break} that decreases with increasing V (Figure 4B). This suggests that tube scission involves thermal activation over a barrier that is lowered by the applied force.

To model this, we assume heterogeneous membrane pore nucleation, occurring at the boundary between the bare tube and the scaffold [1]; once the pore size reaches the scaffold radius r_{s}, scission occurs. Pore nucleation involves passing an energy barrier, which in the context of a membrane tube subject to a force f is given by the change in the thermodynamic function (Landau and Lifshitz, 1986)

$$\Phi = F_{\text{tube}} - fL \qquad \text{S27}$$

for a segment of bare tube of length L . Once a pore forms, assumed to be semi-circular with radius a, rupture costs energy because a membrane edge is exposed; this cost is equal to $\gamma\pi a$, where γ is the edge tension (Evans et al., 2003). In addition, upon pore formation the tube must elongate a bit to accommodate the area $\pi a^2/2$. Since the tube area is conserved, the change in length is given by $\Delta L = (\pi a^2/2)/(2\pi r) = a^2/4r$; this contributes to $\Delta\Phi$ a term $-f\Delta L = -fa^2/4r = -f^2a^2/(8\pi\kappa)$,

[1] Heterogeneous, as opposed to homogeneous, nucleation requires exposing less free membrane edge, thus costing less energy.

where Eq. S10a has been used. Finally, also because of area conservation and because the tube radius r is constant (since f is assumed constant), the bending contribution to $F_{\rm tube}$ is unchanged. Therefore, the barrier for nucleating a pore of size a, which we denote $W_a = \Delta\Phi$, is given by

$$W_a(t) = \gamma\pi a - \frac{f(t)^2 a^2}{8\pi\kappa}\,. \tag{S28}$$

Note that, in principle, the pore radius is variable and the probability of nucleating one with radius a depends on W_a. At a given value of f, as usual in nucleation theory, W has a maximum for a given $a = a_{\rm c}$, and in our case occurs at $a = a_{\rm c} = 4\pi^2\kappa\gamma/f^2$: pores with $a < a_{\rm c}$ re-seal, while those with $a > a_{\rm c}$ grow. We note that for bare membranes $\gamma \sim 10$ pN (Evans et al., 2003), but is significantly reduced in the presence of proteins; see, for example, (García-Sáez et al., 2007; Lee et al., 2008) . Thus, assuming $\gamma = 3$ pN, and $f = 30$ pN, we find that $a_{\rm c} \approx 25$ nm, and thus greater than $r_{\rm s} \approx 10$ nm. This means that pores that spontaneously form with $a < r_{\rm s}$ will not grow and lead to scission, and the only scission-relevant pores are those with radius $r_{\rm s}$. Therefore, the relevant energy barrier is

$$W(t) = \gamma\pi r_{\rm s} - \frac{f(t)^2 r_{\rm s}^2}{8\pi\kappa}\,. \tag{S29}$$

In the following, we separate the force-dependent and independent parts of W as $W = W_0 + W_f$, where

$$W_0 = \pi r_{\rm s}\gamma \tag{S30}$$

and

$$W_f(t) = -\frac{r_{\rm s}^2\, f(t)^2}{8\pi\kappa}\,. \tag{S31}$$

We next calculate the tube scission (or rupture) probability from W and use it to determine $t_{\rm break}$ and the breaking force, $f_{\rm break}$, as functions of V.

3.2 Scission probability

To calculate the scission probability, we apply Kramers' theory for thermally activated processes (Kramers, 1940). Applying this theory, valid for large barriers compared with $k_{\rm B}T$ ($k_{\rm B}$ is Boltzmann's constant and

T is temperature), the force-dependent pore nucleation rate, $\nu(t)$, can be written

$$\nu(t) = \nu_0 \exp\left(-\frac{W_f(t)}{k_{\mathrm{B}}T}\right) . \tag{S32}$$

In the above, $\nu_0 = \bar{\nu}\exp\left(-\frac{W_0}{k_{\mathrm{B}}T}\right)$, where $\bar{\nu}$ is a constant that depends on edge tension, thermal energy, and hydrodynamic drag and is on the order of 10^6 Hz (Evans et al., 2003). Following the general arguments in (Evans et al., 1991; Evans and Ritchie, 1997; Evans et al., 2003), the probability that rupture occurs between t and $t + dt$ is

$$P(t) = \nu(t)\exp\left[-\int_0^t \nu(t')dt'\right] , \tag{S33}$$

where $\exp\left[-\int_0^t \nu(t')dt'\right]$ is the probability that the tube has remained intact up to time t.

The probability $P(t)$ allows us to calculate t_{break}, which we identify with the most probable value of $P(t)$. Noting that $P(t)$ can be expressed as $P(t) = \exp\left[\ln\nu(t) - \int_0^t \nu(t')dt'\right]$, $dP/dt = 0$ occurs at a time t obtained by solving

$$\left.\frac{d\ln\nu}{dt}\right|_{t=t_{\mathrm{break}}} = \nu(t_{\mathrm{break}}) . \tag{S34}$$

Using Eqs. S29 and S32 the above can be re-written as

$$\frac{2}{\nu_0\bar{f}^2} f\dot{f} = e^{f^2/\bar{f}^2} \qquad \text{at } t = t_{\mathrm{break}} , \tag{S35}$$

where $\bar{f}^2 = 8\pi\kappa/r_{\mathrm{s}}^2$. Thus, by solving Eq. S35 using the explicit expression for $f(t)$, Eq. S21, t_{break} can be found, as well as the breaking force $f_{\mathrm{break}} = f(t_{\mathrm{break}})$. These operations determine the scission statistics as a function of V. Generally, Eq. S35 must be solved numerically, though as we calculate below, fairly simple asymptotic expressions for t_{break} and f_{break} can be obtained in limiting cases.

3.3 Expressions for breaking time and force

As represented in the inset of Figure 4B, analytical expressions for t_{break} and f_{break} can be obtained for the two force regimes, $t_{\mathrm{break}} > t^*$ and $t_{\mathrm{break}} < t^*$. For $t_{\mathrm{break}} \gg t^*$, that is, for low V (made more precise below), f is approximately saturated, thus $f = f_{\mathrm{break}} \simeq f_\infty$. Noting that Eq. S21 can be written

$$f(t) = f_\infty \frac{1 + t/t^*}{\left[1 + (t/t^*)^3\right]^{1/3}} , \tag{S36}$$

in the low V regime the time derivative in Eq. S35 is $df/dt \simeq f_\infty t^{*3}/t^4$. Therefore, Eq. S35 becomes

$$\frac{2f_\infty^2}{\nu_0 \bar{f}^2}\frac{t^{*3}}{t^4} = e^{f_\infty^2/\bar{f}^2}\,, \qquad \text{S37}$$

which yields

$$t_{\text{break}} \simeq \tau \exp\left[-\frac{\pi}{k_B T}\left(\frac{\kappa\xi^2 V^2 r_{\text{s}}^4}{128}\right)^{1/3}\right]\,; \qquad \text{S38}$$

see also Eq. 6 of the main text. In the above equation, the time constant is given by

$$\tau = \frac{2^{7/6}\,\pi\left(\kappa^7\xi^5 r_{\text{s}}\right)^{1/12}}{V^{1/3}}\left(\frac{L_0^3}{f_0^3 k_{\text{B}} T \nu_0}\right)^{1/4}\,. \qquad \text{S39}$$

In the opposite limit, $t_{\text{break}} \ll t^*$, $f \simeq f_0\,(1 + Vt/L_0)$, and therefore Eq. S35 can be written

$$\frac{2V f_0 f}{\nu_0 L_0 \bar{f}^2} = e^{f^2/\bar{f}^2}\,. \qquad \text{S40}$$

Therefore, for large V,

$$f_{\text{break}} \simeq \bar{f}\sqrt{\ln\left(\frac{2V f_0^2}{\nu_0 L_0 \bar{f}^2}\right)}\,. \qquad \text{S41}$$

and

$$t_{\text{break}} = \frac{L_0}{V}\left(\frac{f_{\text{break}}}{f_0} - 1\right)\,. \qquad \text{S42}$$

We note that the crossover value of V between the two regimes is found roughly by equating the expressions in Eq. S38 and S42; see also the inset of Figure 4B. Assuming $\xi = 50$ Pa.s, $f_0 = 10$ pN, $\gamma = 3$ pN, and $L_0 = 1$ μm, we find that this value is $V \approx 1$ μm/s. Since most of our scission data occurs for pulling speeds less than this value, we therefore used Eq. S38 to fit the measured breaking times and Eq. S14 the breaking forces, thereby yielding further estimates of ξ; see Figures 4C, 4E, and 4F.

Note finally that the expressions obtained here for t_{break} and f_{break} depend on $P(t)$ having a narrow peak and on the assumption $\alpha \gg 1$ that underpins Equation S21; see Sec. 2.2. Thus, these expressions break down for small ξ, which can be seen from the unphysical result that $t_{\text{break}} \to 0$ for $\xi \to 0$ in Eq. S38. As argued above in Sec. 2.2, our experimental data indicate that $V' > 1$ and $\alpha \gg 1$, thus validating the approximations leading to the expressions in this section.

3.4 Note on other scission mechanisms

We have considered above a model of tube scission that depends on membrane pore nucleation. Two other routes to scission are possible, which we discuss below. We show that these are not applicable to BAR protein-mediated tube scission, which occurs on a seconds-to-minutes time scale.

First, local tube pinching from a radius r_0 down to $r_i \approx 3$ nm can lead to scission via a hemi-fission intermediate state (Kozlovsky and Kozlov, 2003). The corresponding energy barrier is $\Delta E \approx \pi^2\kappa^2 \left(r_i^{-1} - r_0^{-1}\right)/f$ (Morlot et al., 2012). In the case of dynamin-assisted scission, GTP hydrolysis constricts the dynamin coat down to $r_0 \approx 4.5$ nm, thereby significantly lowering the energy barrier. In contrast, r_0 for endoA2-scaffolded tubes is much larger: the scaffold radius itself is 10 nm (Simunovic et al., 2016; Renard et al., 2015), and the adjacent bare membrane tube, even at the highest attained forces, around 70 pN, has a radius no smaller than ∼15 nm; see Eq. S10a. Thus, in our case, $\Delta E \approx 250\ k_\mathrm{B}T$. This energy corresponds to a scission time of $\tau \exp\left(\Delta E/k_\mathrm{B}T\right) \approx 10^{96}$ hours, where $\tau \sim 10^{-9}$ s (Morlot et al., 2012), and thus impossible!

Secondly, it has been proposed that line tension, which arises at the boundary between lipid domains and acts to reduce the boundary length, could constrict tubes enough to cause scission (Allain et al., 2004; Römer et al., 2010). For tubes that are partially coated by a BAR domain scaffold, Liu et al. proposed that sequestration of PI(4,5)P2 by the scaffold results in a line tension at the interface with the bare membrane (Liu et al., 2009). In contrast with their model of endocytosis, in which enzymatic activity amplifies PI(4,5)P2 concentration differences, in our case the PI(4,5)P2 enrichment under the coat is limited to a factor of three (Picas et al., 2014), and thus, according to (Liu et al., 2009), a line tension of $\lambda \approx 5$ pN. As a result, tube scission would release an energy $2\pi\lambda r_\mathrm{s} \approx 78\ k_\mathrm{B}T$, and our above estimation for the energy barrier would be reduced to $170\ k_\mathrm{B}T$. This barrier is still too great to be passed over by thermal processes on any reasonable timescale, and we thus rule it out.

4 Note on why different measurements yield different values of the friction coefficient

Here, we briefly comment on the different ways to estimate the protein/lipid friction coefficient, ξ (Table S2), and why these lead to different values. The first way of estimating ξ was from individual fits to the force versus time data sets (Table S2, first row). For each protein type (endoA2 WT, endoA2 mut, and

endoA2 ΔH0), a number n of data sets were fitted, from each of which a value of ξ was obtained through Eq. S21. We note that each f vs t set comes from a single pulling experiment, with a given protein scaffold (and thus given length, L_{s}). The reported value of ξ for each protein was the average over the n fitted data sets.

In contrast, the values of ξ given in the second and thirds rows of Table S2 were obtained from fits to the scission data set (force at breakage versus V and time until breakage vs. V), each point in the set corresponding to a different pulling experiment. As a result, the manner in which the data were averaged is not the same as described above. (We note, however, that the values obtained from f_{break} vs. V and t_{break} vs. V are within the margin of error for endoA2 WT). These two different averaging methods result in different values of ξ values because of variability in scaffold properties from one experiment to another. This is one reason for the discrepancy in the values of ξ for a given protein type between the different estimation methods.

A second reason for the discrepancy could be related to our model of pore nucleation leading to scission. Namely, we assume that a semi-circular pore of radius equal to that scaffold nucleates at the scaffold/bare tube interface. Considering the tubular geometry, this might be an approximation to reality; nevertheless, a careful description of the pore shape is beyond the scope of this manuscript.

5 Note on sources of protein/lipid friction and tension along tube

We briefly justify here the expression for the tube tension as a function of lipid velocity, Eq. S7. If we consider the force balance on a cylindrical element of lipid bilayer, of constant radius, underneath the protein scaffold, we obtain

$$\frac{\partial \sigma}{\partial z} = \zeta v_l \,, \qquad \text{S43}$$

where ζ is an intensive friction coefficient and σ is the local tension. Integrating this expression from $z = 0$ (base of the scaffold, assumed to coincide with tube neck) to $z = L_{\mathrm{s}}$ (scaffold length), we recover Eq. S7, where we identify ξ as ζL_{s} and σ_0 as the tension on the vesicle.

There is, however, another source of protein/lipid friction that complicates the above picture. Namely, since the scaffold is anchored to the GUV (note Figure 1B, for example, where the scaffold is seen to move with the displaced GUV), there must be additional dissipation. This most likely comes from friction between lipids and proteins at the tube neck, and possibly over an extended part of the GUV. As

a result, we expect that ξ can be written $\xi = \zeta L_{\rm s} + \xi'$, where ξ' is independent of the scaffold length. It is difficult to estimate ξ', though one line of reasoning goes as follows. As the tube is pulled, the neck presents a barrier of width $\sim r_{\rm s}$ to tube-directed lipids from the GUV. Since ξ' is dimensionally given by a force per unit area multiplied by time, at the scaling level, it can be estimated as the force per unit area acting on lipids as they pass the neck, times the barrier crossing time. Thus,

$$\xi' \sim \frac{W_{\rm n}}{e^2 r_{\rm s}} \tau_0 \, e^{\frac{W_{\rm n}}{k_{\rm B}T}} \,, \qquad \text{S44}$$

where e is the bilayer thickness, τ_0 is the lipid diffusion time over a distance $r_{\rm s}$, and $W_{\rm n}$ is the barrier height. Taking the lipid diffusion constant $D = 10^{-12}$ m^2/s, $r_{\rm s} = 10$ nm, and $e = 5$ nm, we obtain $\xi' \sim$ (1 Pa.s) $W_{\rm n}/(k_{\rm B}T)\exp(W_{\rm n}/k_{\rm B}T)$. Thus, for ξ' to be of the same order as $\zeta L_{\rm s}$ ($\approx$ 50 Pa.s), the barrier height need only be on the order of a few $k_{\rm B}T$. This barrier height is not that high, and this argument likely explains why assigning all the friction to $\zeta L_{\rm s}$ is inaccurate.

Finally, we point out recent work suggesting that the stiffness and spontaneous curvature of a localized protein patch influence the tension on a membrane (Rangamani et al., 2014; Walani et al., 2015; Hassinger et al., 2017). These studies consider the mechanics of a composite protein plus lipid membrane with inhomogeneous material properties. Recall that in our experiments, the protein scaffold is assumed to be a fixed, rigid cylindrical coat that does not change in time. As a result, in the model we only consider the dynamics of the lipid bilayer flowing under this fixed coat, and relate its tension to the lipid flow. Even if the scaffold's bending ridigity and intrinsic curvature affect the tension, according to Ref. (Rangamani et al., 2014), since the scaffold's material properties are assumed to remain constant in time, the influence on tension does not change with time, and cannot explain the tension "build-up" that can be inferred from the tube pulling force, generated externally. Thus, in our case, it is not necessary to use a formalism, such as presented in Ref. (Rangamani et al., 2014), that focuses on the composite membrane (proteins and lipids together).

This is in contrast with Ref. (Hassinger et al., 2017), in which a composite membrane description is appropriate, since the coat formed by clathrin proteins is not fixed, and for which the shape of the protein plus lipid membrane evolve together as a result of localized spontaneous curvature and bending rigidity.

Supplemental references

García-Sáez, A. J., Chiantia, S., Salgado, J. and Schwille, P. (2007). Pore formation by a Bax-derived peptide: effect on the line tension of the membrane probed by AFM. Biophys J *93*, 103–12.

Hassinger, J. E., Oster, G., Drubin, D. G. and Rangamani, P. (2017). Design principles for robust vesiculation in clathrin-mediated endocytosis. Proc Natl Acad Sci U S A *114*, E1118–E1127.

Keller, J. B. and Rubinow, S. I. (1976). Slender-body theory for slow viscous flow. Journal of Fluid Mechanics *75*, 705–714.

Landau, L. D. and Lifshitz, I. L. (1986). Statistical Physics, Part 1. 3rd edition, Butterworth Heinemann, London.

Lee, M.-T., Hung, W.-C., Chen, F.-Y. and Huang, H. W. (2008). Mechanism and kinetics of pore formation in membranes by water-soluble amphipathic peptides. Proceedings of the National Academy of Sciences *105*, 5087–5092.

Liu, J., Sun, Y., Drubin, D. G. and Oster, G. F. (2009). The mechanochemistry of endocytosis. PLoS Biol *7*, e1000204.

Picas, L., Viaud, J., Schauer, K., Vanni, S., Hnia, K., Fraisier, V., Roux, A., Bassereau, P., Gaits-Iacovoni, F., Payrastre, B., Laporte, J., Manneville, J.-B. and Goud, B. (2014). BIN1/M-Amphiphysin2 induces clustering of phosphoinositides to recruit its downstream partner dynamin. Nat Commun *5*, 5647.

Rangamani, P., Mandadap, K. K. and Oster, G. (2014). Protein-induced membrane curvature alters local membrane tension. Biophys J *107*, 751–62.

Walani, N., Torres, J. and Agrawal, A. (2015). Endocytic proteins drive vesicle growth via instability in high membrane tension environment. Proc Natl Acad Sci U S A *112*, E1423–32.

Table S1. Statistics of scission events. Related to Figures 1, 3, and 4. Shown is number of spontaneously observed scission events after injecting the protein near the pulled tube and by elongation of a protein-scaffolded tube. Average breakage force and time are shown for the case of one pulling velocity. Both the full-length endoA2 and just its N-BAR domain stabilized the tubes and induced scission upon elongation and were pooled in the statistics. The total number of experiments is given in brackets. EndoA2, endophilin A2 WT; endoA2 ΔH0, endophilin A2 with truncated N-terminal helices; endo mut, endophilin A2 E37K, D41K; centaurin, β2 centaurin; *V*, tube extension velocity.

	Spontaneous scission events	Scission events by tube extension in the range V = 50–8000 nm.s^{-1}	Average breakage force, Δf, at V = ~0.5 µm.s^{-1}	Average breakage time at V = ~0.5 µm.s^{-1}
endoA2	3 (72)	40 (43)	31±1 pN (5)	25±11 s (5)
endo ΔH0	0 (6)	6 (6)	-	-
endo mut	0 (13)	12 (13)	-	-
centaurin	1 (16)	5 (8)[a]	20±8 pN (3)	92±30 s (3)

[a]In the three negative cases, the bead was ejected from the trap.

Table S2. Friction coefficients. Related to Figures 3 and 4 and STAR Methods. Scaffold-lipid friction coefficients for endoA2 WT and its mutants. See STAR Methods for details on measurements and potential source of errors related to the measurement type.

Measurement type	ξ_{WT} (Pa.s)	$\xi_{\Delta H0}$ (Pa.s)	ξ_{mut} (Pa.s)
Force *vs*. time	80±30	39±19	112±27
Breaking forces	30±12	1.4±2	66±6
Breaking times	56±16	-	-
