# Supplementary figures and images for: Friction Mediates Scission of Tubular Membranes Scaffolded by BAR Proteins
Source: Cell. 2017 Jun 29;170(1):172–184.e11. doi: 10.1016/j.cell.2017.05.047 (PMC5576516; doi:10.1016/j.cell.2017.05.047)

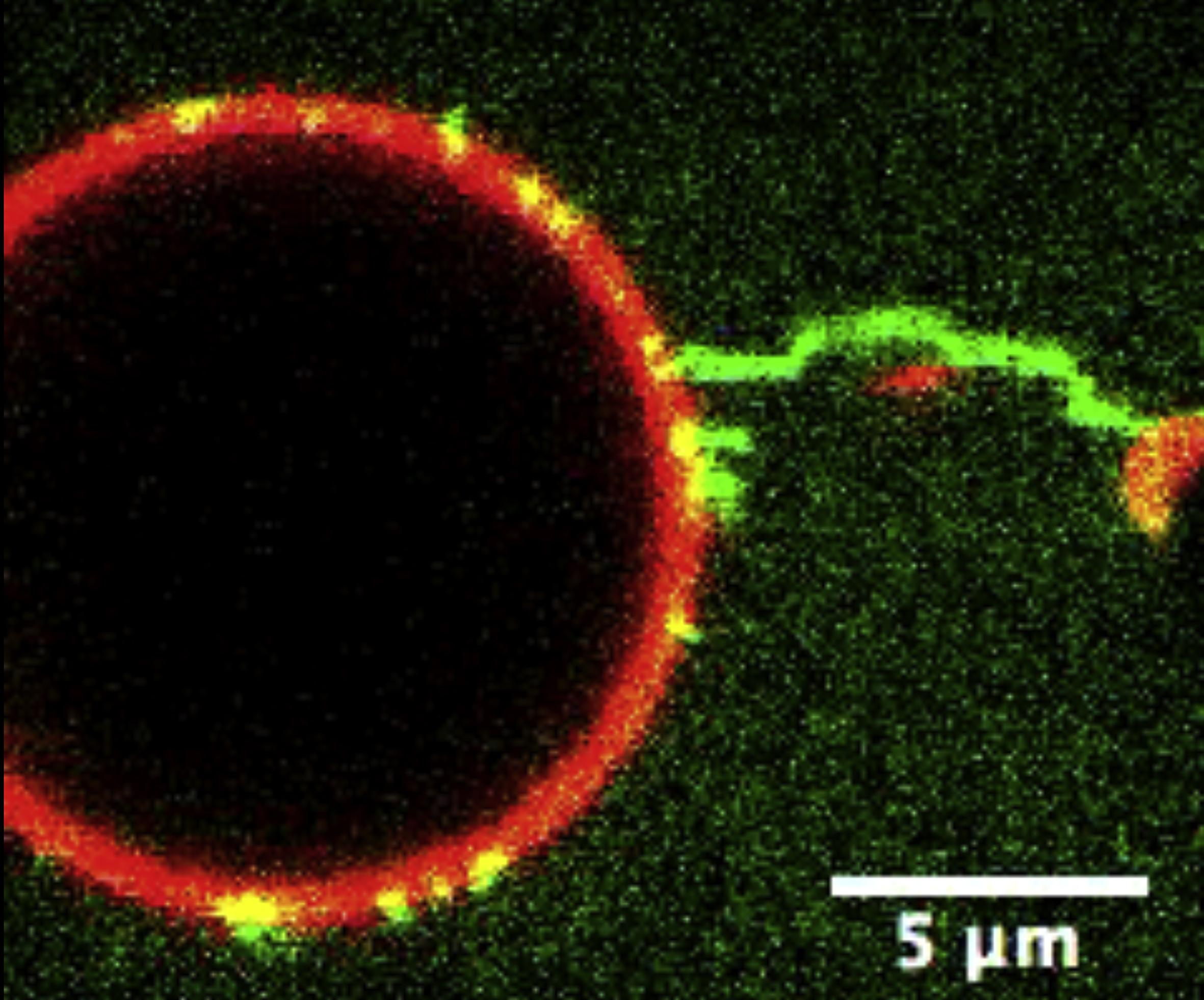

Supplement: Movie S1. EndoA2 N-BAR Domain Does Not Induce Scission of Tubular Membranes, Related to Figure 1 and Figure S1 — Confocal fluorescence time-lapse during injection of an N-BAR domain of endoA2 near a tube pulled from a micropipette-aspired GUV. Movie shows spontaneous tubulation of the GUV and the formation of a scaffold on the tube (causing it to buckle). No scission is observed under these conditions. Red, lipids; green, NBAR domain. [file mmc2.jpg]

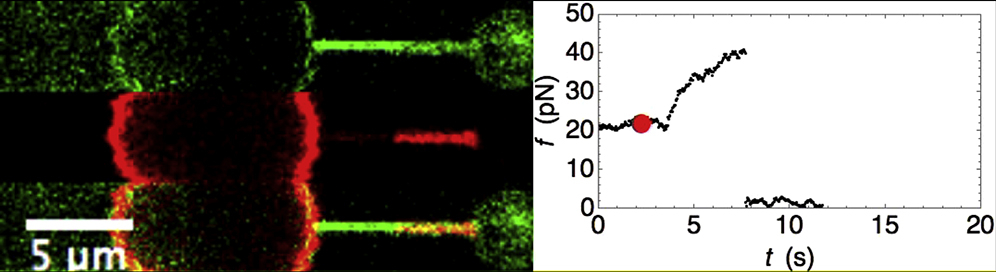

Supplement: Movie S2. FDS of a Tube Partially Scaffolded by EndoA2, Related to Figure 1 — Movie shows extension of a tube partially scaffolded by endoA2 N-BAR domain leading up to scission at the tube-GUV interface. Shown are the confocal fluorescence time lapse (left) and the tube retraction force, f, both changing with time, t. Top left: N-BAR (green); center left: lipids (red); bottom left: overlay [file mmc3.jpg]

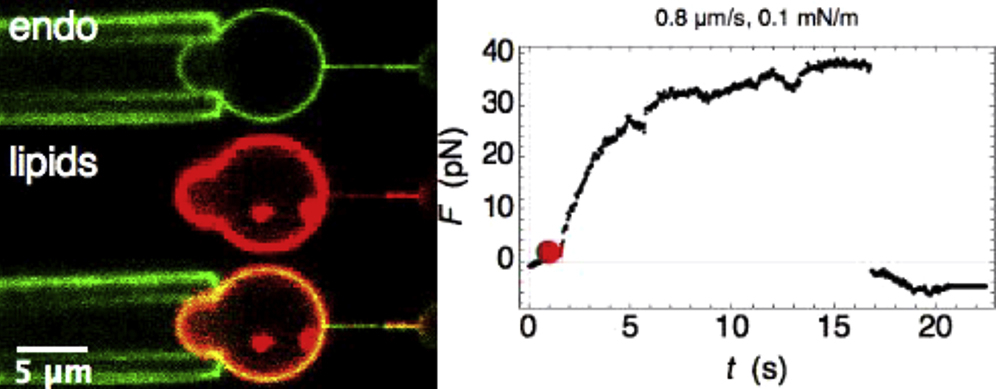

Supplement: Movie S3. FDS of a Tube Fully Scaffolded by EndoA2, Related to Figure 1 — Movie shows extension of a tube initially fully scaffolded by endoA2 N-BAR domain leading up to scission. After initial extension, gaps form in the scaffold making fully and partially scaffolded tubes equivalent in FDS. Shown are confocal fluorescence time lapse (left) and tube retraction force, f, both changing with time, t. Top left: N-BAR (green); center left: lipids (red); bottom left: overlay [file mmc4.jpg]

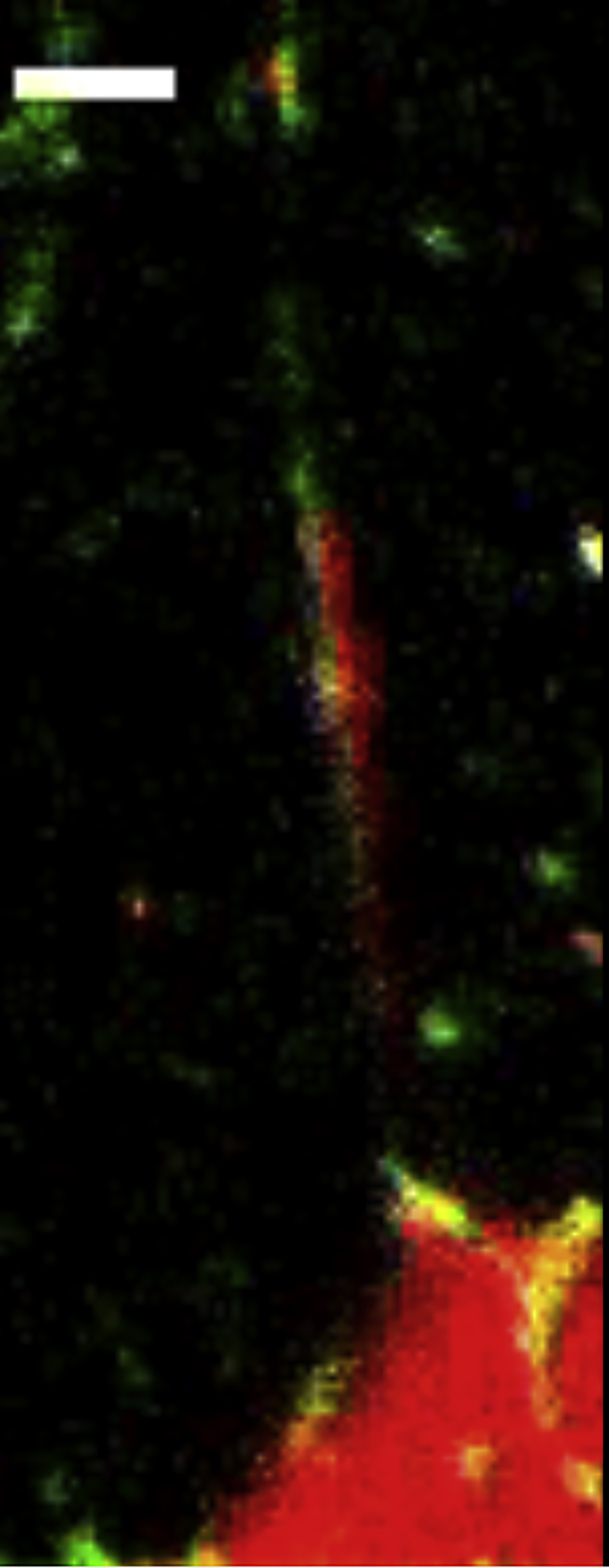

Supplement: Movie S4. FDS by Kinesin, Related to Figure 5 and Figure S6 — Confocal fluorescence time lapse showing two scission events of kinesin-pulled tubes connected to a GUV seconds after endoA2 injection into the system. Scale bar, 2 μm. [file mmc5.jpg]

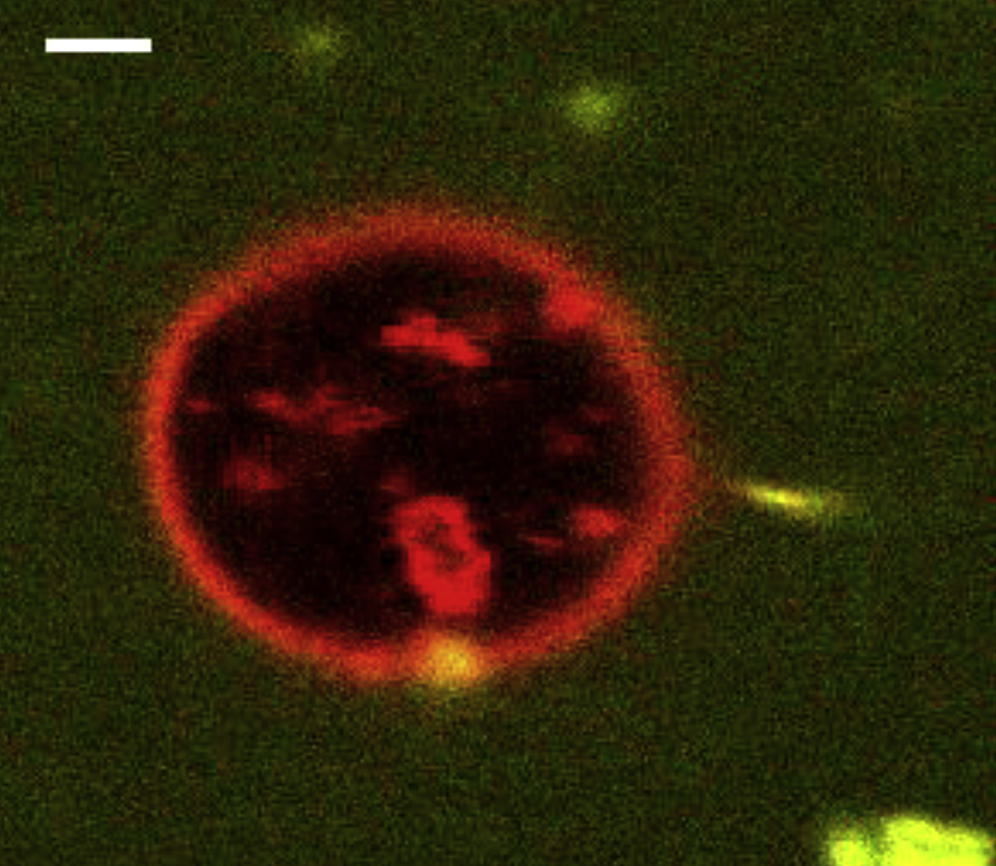

Supplement: Movie S5. FDS by Kinesin, Related to Figure 5 and Figure S6 — Confocal fluorescence time lapse showing a scission event of a kinesin-pulled tube connected to a GUV seconds after endoA2 injection into the system. Shown is a different example from Movie S4. Scale bar, 2 μm. [file mmc6.jpg]
